# Polylactic acid-based dressing with oxygen generation and enzyme-like activity for accelerating both light-driven biofilm elimination and wound healing

**DOI:** 10.1093/burnst/tkae041

**Published:** 2024-10-25

**Authors:** Tianci Wen, Shilang Xiong, Huihui Zhao, Junzhe Wang, Chunming Wang, Zhisheng Long, Long Xiong, Guowen Qian

**Affiliations:** School of Energy and Mechanical Engineering, Jiangxi University of Science and Technology, No. 1180, Shuanggang East Street, Qingshanhu District, Nanchang City, Jiangxi Province 330013, P.R. China; Department of Orthopedics, The First Affiliated Hospital of Nanchang University, Artificial Joints Engineering and Technology Research Center of Jiangxi Province, Nanchang 330006, P.R. China; School of Energy and Mechanical Engineering, Jiangxi University of Science and Technology, No. 1180, Shuanggang East Street, Qingshanhu District, Nanchang City, Jiangxi Province 330013, P.R. China; School of Energy and Mechanical Engineering, Jiangxi University of Science and Technology, No. 1180, Shuanggang East Street, Qingshanhu District, Nanchang City, Jiangxi Province 330013, P.R. China; School of Energy and Mechanical Engineering, Jiangxi University of Science and Technology, No. 1180, Shuanggang East Street, Qingshanhu District, Nanchang City, Jiangxi Province 330013, P.R. China; Department of Orthopedics, Jiangxi Provincial People's Hospital, The First Affiliated Hospital of Nanchang Medical College, No. 152, Aiguo Road, Donghu District, Nanchang City, Jiangxi Province 330006, P.R. China; Department of Orthopedics, The Second Affiliated Hospital of Nanchang University, No. 1 Minde Road, Donghu District, Nanchang City, Jiangxi Province 330008, P.R. China; School of Energy and Mechanical Engineering, Jiangxi University of Science and Technology, No. 1180, Shuanggang East Street, Qingshanhu District, Nanchang City, Jiangxi Province 330013, P.R. China

**Keywords:** Nanozyme, Photodynamic therapy, Anti-biofilm, Infectious wound repair, Electrospinning, Manganese dioxide, Dressing

## Abstract

**Background:**

Photodynamic therapy (PDT) is a widely used therapeutic approach for eradicating bacterial biofilms in infected wound, but its effectiveness is limited by the hypoxic environment within the biofilm. This study aimed to investigate whether the efficiency of photodynamic removing biofilm is improving by providing oxygen (O_2_), as well as the expression of cytokines involved in infected wound healing.

**Methods:**

Manganese dioxide (MnO_2_) nanoparticles with catalase-like activity were grown *in situ* on graphitic phase carbon nitride (g-C_3_N_4_, CN) nanosheets to construct an all-in-one CN-MnO_2_ nanozyme, which was then incorporated into poly-L-lactic acid (PLLA) to prepare CN-MnO_2_/PLLA wound dressing by electrospinning. Subsequently, the *in vitro* antibacterial biofilm ratio and antibacterial ratio of CN-MnO_2_/PLLA wound dressing were examined by spread plate and crystal violet staining under irradiation with 808 nm near-infrared light and 660 nm visible light. Meanwhile, the rat skin injury model was established, and hematoxylin and eosin (H&E), Masson’s, tumor necrosis factor-α (TNF-α), Arginase 1 (Arg-1), vascular endothelial growth factor (VEGF) and basic fibroblast growth factor (BFGF) were evaluated *in vivo* to assess the effect of CN-MnO_2_/PLLA wound dressing on wound healing.

**Results:**

Biofilm density caused by *Staphylococcus aureus* and *Pseudomonas aeruginosa* had elimination rates of 83 and 62%, respectively, when treated with CN-MnO_2_/PLLA dressing. Additionally, the dressing exhibited high antibacterial efficacy against both bacteria, achieving 99 and 98.7% elimination of *Staphylococcus aureus* and *Pseudomonas aeruginosa*, respectively. Furthermore, *in vivo* experiments showed that the CN-MnO_2_/PLLA wound dressing achieved complete healing of infected wounds on Day 14, with a wound healing rate of >99% by increasing collagen deposition, expression of anti-inflammatory cytokine Arg-1, vascularization cytokine VEGF, and epithelial cell BFGF, and inhibiting the expression of inflammatory cytokine TNF-α.

**Conclusions:**

The CN-MnO_2_/PLLA wound dressing exhibited excellent antibacterial properties *in vitro* and *in vivo*. In addition, CN-MnO_2_/PLLA wound dressing accelerated rapid wound healing through an anti-inflammatory, pro-vascular regeneration and skin tissue remodeling mechanism.

## Graphical Abstract

**Graphical Abstract:**
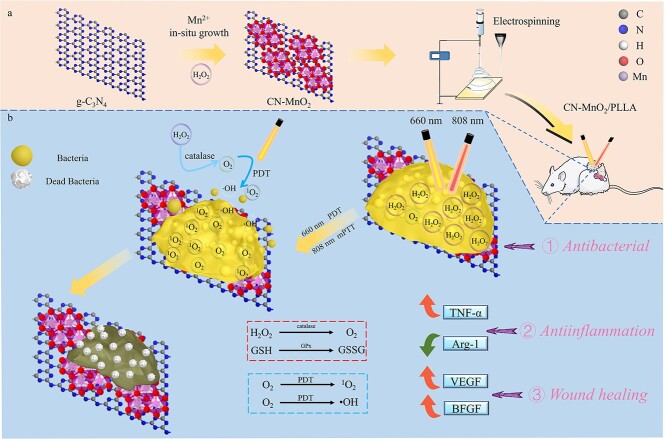

Highlights

- CN-MnO_2_/PLLA was prepared by the electrospinning method, which solved the problem of insufficient O_2_ in the biofilm limiting PDT, by consuming hydrogen peroxide inside the biofilm to produce O_2_, and provides a new method for treating persistent bacterial infections.
- Dual-light irradiation of CN-MnO_2_/PLLA to generate reactive oxygen species and mild photothermal therapy effects was applied to wound treatment, and it was proved that CN-MnO_2_/PLLA wound dressings were efficiently antibacterial both *in vitro* and *in vivo*.
- It was proved that CN-MnO_2_/PLLA achieves an anti-inflammatory, pro-vascularization, and pro-epithelialization effect to accelerate wound healing by inhibiting the expression of TNF-α and promoting the expression of Arg-1, VEGF, and BFGF.

## Background

Trauma, infection, burns, and diabetes are common causes of full-layer skin defects [1]. The use of wound dressings for repairing such defects has brought about a new frontier in skin wound healing. However, bacteria frequently cause abscesses on skin wounds and develop dense bacterial biofilms in the clinic, leading to delays or failures in wound healing. Moreover, bacteria produce hydrogen peroxide (H_2_O_2_) in the process of biofilm formation, which weakens the immune system and the effects of antibiotics and immune cells. The formation of biofilms easily causes persistent bacterial infections and seriously threatens the life and health of patients [2,3]. Therefore, it is imperative to develop an effective strategy to eliminate bacterial biofilms.

Substantial efforts have been dedicated to developing new antibacterial materials, such as antimicrobial peptides and heavy-metal ions, to protect the public against bacterial infections [4–6]. However, the high cost and potential biological toxicity of these materials limit their practical application. Photodynamic therapy (PDT) is a highly promising antibacterial strategy that utilizes photosensitizers to generate reactive oxygen species (ROS) such as singlet oxygen (^1^O_2_), hydroxyl radicals (•OH), and superoxide anions (${\mathrm{O}}_2^{\bullet -}$) upon exposure to a specific wavelength of irradiation [7–9]. PDT has been widely noted for its ability to destroy the biofilm matrix and kill bacteria with less harm to normal tissues [9,10]. Among all types of photosensitizers, 2D graphitic phase carbon nitride (g-C_3_N_4_, CN), consisting of tri-s-triazine units bridged with tertiary nitrogen, has great application prospects in PDT due to its excellent biocompatibility, good stability, and moderate band gap (2.7 eV). However, an insufficient O_2_ supply limits the generation of ROS within bacterial biofilms. Thus, it is highly desirable but challenging to simultaneously achieve a sufficient O_2_ supply and ROS generation to overcome hypoxia-associated resistance.

In recent years, researchers have performed various studies to overcome hypoxia within biofilms. Catalase can decompose endogenous H_2_O_2_ in bacterial biofilms to generate O_2_, which has a significant effect on the enhancement of the photodynamic effect [11]. Manganese dioxide (MnO_2_), as a nanozyme_,_ possesses excellent catalase-like activity, and can consume H_2_O_2_ and achieve a self-supply of O_2_ [12]. Excitingly, MnO_2_ displays strong light absorption in the near-infrared (NIR) light region, which can generate mild photothermal effects under NIR light irradiation [13]. Mild photothermal therapy (mPTT) can increase the permeability of biofilms and enhance catalase activity [14]. Moreover, MnO_2_ also has glutathione peroxidase (GPx) activity and consumes glutathione (GSH) in bacteria, thus weakening the bacterial antioxidant system. Additionally, several studies have demonstrated that the *in situ* growth of MnO_2_ on the surface of CN nanosheets effectively inhibits the recombination of electron–hole pairs and enhances the production of ROS [15].

Herein, a CN-MnO_2_ nanozyme was constructed by *in situ* growth of MnO_2_ nanoparticles with catalase activity on the surface of CN nanosheets, which were then added to poly-L-lactic acid (PLLA) to fabricate a CN-MnO_2_/PLLA wound dressing by electrospinning. The dressing could simultaneously achieve PDT and mPTT under dual-light irradiation. MnO_2_ continuously catalyzes H_2_O_2_ to generate O_2_. This O_2_ can further be converted into ^1^O_2_ and •OH by CN under visible light (VL) irradiation. The mild photothermal effect of MnO_2_, when exposed to NIR light, can enhance the sensitivity of biofilms and their catalase activity. Significantly accelerated wound healing is achieved by inhibiting the growth of bacteria, decreasing the expression of pro-inflammatory cytokine (TNF-α) and accelerating anti-inflammatory cytokine (Arg-1), vascular endothelial growth factor (VEGF), and basic fibroblast growth factor (BFGF) expression. We characterized the morphology, microstructure, and constituents of the synthesized CN-MnO_2_ nanozymes. Additionally, we systematically investigated the photodynamic effect, photothermal property, enzyme-like activity, antibacterial property, antibiofilm activity, and *in vivo* infectious wound repair effect of the prepared CN-MnO_2_/PLLA wound dressing.

## Methods

### Raw materials

Melamine (C_3_H_6_N_6_ 99%, Shanghai Aladdin), manganese chloride (MnCl_2_·4H_2_O 99%), tetramethylammonium hydroxide solution (TMAOH, 10 wt. % in methanol) and H_2_O_2_ solution (30 wt.% in H_2_O) were purchased from Shanghai Aladdin Biochemical Technology Co., Ltd. Poly-L-lactic acid (MW = 150 kDa) powders were bought from Shenzhen Polymtek Biomaterial Co., Ltd. The chemicals do not need further purification for use.

### Preparation of g-C_3_N_4_ nanosheets

Melamine powder (5 g) was poured into an alumina crucible that was then covered with a lid. The crucible was placed in a muffle furnace (PD-MJ14, Luoyang) for high-temperature calcination, and the heating rate was set to 5°C/min. The temperature in the furnace was increased from room temperature to 550°C and kept at that temperature for 4 h. After cooling to room temperature, the crucible was removed from the furnace and a faint yellow powder was obtained. After grinding, the powder was calcined at 550°C again for 4 h to obtain fine graphene-phase carbon nitride (g-C_3_N_4_, CN) nanosheets.

### Preparation of CN-MnO_2_ nanozyme

Firstly, 200 mg of CN powder was added to 30 ml of deionized (DI) water and sonicated for 30 min. MnCl_2_·4H_2_O (148.5 mg) was dissolved in 40 ml of DI water. Then, 6 ml of TMAOH and 1.5 ml of 60 mM H_2_O_2_ solution were added into the above solution, and stirred slowly for 12 h. Subsequently, the brown precipitate was collected after centrifugation at 7000 rev/min for 5 min. After washing three times with DI water and three times with methanol, the CN-MnO_2_ nanozyme was obtained via a freeze-dry method.

### Characterization of CN-MnO_2_ nanozyme

The morphology and microstructure of the CN-MnO_2_ nanozyme were analyzed using a transmission electron microscope (FEI Tecnai F20, USA). The chemical functional groups of the CN-MnO_2_ were examined by Fourier-transform infrared (FTIR) spectrometry (FTIR-8050, Tian Jin Gang Dong Sci.&Tech, China). The phase of the CN-MnO_2_ was measured with an X-ray diffraction (XRD) instrument (D8 Advance, Bruker Co, Germany). The chemical composition of the CN-MnO_2_ was measured using X-ray photoelectron spectroscopy (XPS; Thermo, USA). The visible-NIR absorption spectra of the CN-MnO_2_ were measured at 400–900 nm using a microplate reader (Varioskan LUX, Thermo Scientific).

### Preparation of wound dressings

PLLA powder (700 mg) was dissolved in the 10 ml of dichloromethane/*N*,*N*-dimethyl formamide (v/v = 4 : 1) mixed solution and stirred continuously for 12 h. The PLLA spinning solution was poured into a 5 ml plastic syringe equipped with a blunt 19-gauge needle (inner diameter 0.7 mm). The needle was connected to a high-voltage power supply, and a voltage of 20 kV was applied. The spun filaments were collected using tinfoil placed at a distance of 4 cm from the tip of the needle. The PLLA wound dressing was successfully prepared with a pushing speed of 0.005 mm/s at 35°C and 40% humidity. In addition, The CN/PLLA and CN-MnO_2_/PLLA wound dressings were prepared by adding 123 mg of CN and 123 mg of CN-MnO_2_ to 700 mg of PLLA powder for electrospinning, respectively. If not specified otherwise in the subsequent experiments, the dimensions of the dressing were set as 1 × 1 cm squares.

### Physicochemical properties of the wound dressings

#### Morphology observation

The morphology of the wounds dressings was observed using scanning electron microscopy (SEM; EVO18, ZEISS, Germany). The diameters of 100 fibers were randomly selected and calculated using ImageJ software.

### Porosity determination

The porosity of the wound dressing was tested using a weighing method. The initial dressing was cut to 5 × 2 cm and weighed to obtain an original mass of *W*_0_. Subsequently, the dressing was completely immersed in phosphate buffer solution (PBS) for 24 h. The dressing was taken out, and excess PBS on its surface was gently absorbed using absorbent paper. The weight of the wet dressing was measured and recorded as *W*_1_. Then the wet dressing was dried in an oven at 80°C for 3 h. The weight of the dry dressing was defined as *W*_2_. The porosity of the dressing was calculated using the formula: porosity (%) = *V*_pore_/(*V*_pore_ + *V*), where *V*_pore_ = (*W*_1_−*W*_2_)/ρ_PBS_, and *V* = *W*_0_/ρ_PLLA_; where *V*_pore_ represent the pore volume of the wound dressing, *V* represent the volume of the wound dressing, ρ_PBS_ is the density of PBS, and ρ_PLLA_ is the density of PLLA.

### Water vapor transmittance and water contact angle

The water vapor transmittance rate (WVTR) of the wound dressing was calculated by a weight method. Distilled water (5 ml) was added to a glass vial. The mouth of the glass bottle was then covered with the wound dressing, which could be either PLLA, CN/PLLA, or CN-MnO_2_/PLLA. The blank group was the open-capped glass vial. The glass vial was put into a drying oven at 30°C. After 24 h of incubation, the weight of the glass vial was measured. Then WVTR was calculated using the following equation: WVTR (%) = (*W*_0_−*W*_t_)/*S* × 100%, where *W*_0_ is the initial weight of glass vial with 5 ml of DI water and wound dressing, *W*_t_ is the weight of glass vial with 5 ml of DI water after 24 h, and *S* is the area of the mouth of the glass vial. The water contact angle (WCA) of the wound dressing surface was measured by a WCA microscope (BX53F2, OLYMPUS, Tokyo, Japan) in the range 0–180° to determine the hydrophilicity of the dressing surface.

### Oxidase activity

3,3′,5,5′-Tetramethylbenzidine (TMB, Beyotime) was used to examine the oxidase activity by a color development reaction. Briefly, 200 μl of TMB solution [240 mg/l, dimethylsulfoxide (DMSO)] was added to 1 ml of CH_3_COOH solution (pH = 4.0, Aladdin). Then, the wound dressings (PLLA, CN/PLLA, and CN-MnO_2_/PLLA) were immersed in the above solution for 30 min. The color of the solution was photographed using a digital camera. Meanwhile, the absorption spectra between 400–800 nm of the above solutions were tested using a microplate reader (Varioskan LUX, Thermo Scientific, USA).

### Peroxidase activity

In addition, the peroxidase activity of the CN-MnO_2_/PLLA wound dressing at different pH values and times was evaluated using TMB. Briefly, 1.5 ml of detection solution, including 892.5 μl of CH_3_COONa (61 mg/ml, DI water), 600 μl of TMB (0.5 mg/ml, DMSO), and 7.5 μl of H_2_O_2_ (4 mM, DI water), was prepared. The CN-MnO_2_/PLLA wound dressing was added into the detection solution and incubated at 37°C at different pHs (5.5, 6.5, and 7.4) for 0, 10, 20, and 30 min. The optical density (OD) value at 652 nm of the above solutions was detected using a microplate reader (Varioskan LUX, Thermo Scientific, USA).

### H_2_O_2_ consumption

Titanium sulfate (TiSO_4_) is used to test the consumption of H_2_O_2_ by a color development reaction. H_2_O_2_ can react with TiSO_4_ to generate a yellow titanium peroxide complex (TiO_2_^2+^). The depth of yellow color is correlated linearly with the concentration of H_2_O_2_. Briefly, the wound dressings (PLLA, CN/PLLA, and CN-MnO_2_/PLLA) were immersed in a 1.5 ml Eppendorf (EP) tube with 1 ml of TRIS buffer solution (10 mM TRIS, 0.17 M NaCl, pH = 5.5) and 5 μl of 4 mM H_2_O_2_. The wound dressings were incubated at 37°C on a shaker at 120 rpm for 0, 10, 20, and 30 min. Afterwards, 90 μl of the mixed solution was taken up, and 10 μl of TiSO_4_ (50 mg/ml) was added and kept for 2 min, and the OD value at 412 nm of the yellow solution was measured using a microplate reader (Varioskan LUX, Thermo Scientific, USA).

### O_2_ generation

O_2_ generation in aqueous solution was measured using a portable dissolved-oxygen meter (JPB-607A, Qiwei Technology Co., Ltd, Hangzhou). Specifically, to assess the O_2_ generation capacity of the wound dressing, 5 μl of 4 mM H_2_O_2_ was added to 1 ml of PBS with the wound dressing. Then, an O_2_ electrode probe was inserted into the EP tube to measure the O_2_ level. O_2_ generation was measured every 10 min under different conditions. O_2_ generation in different groups (blank, PLLA, CN/PLLA, and CN-MnO_2_/PLLA, at 37°C, pH = 5.5), pH (pH = 5.5, 6.5, and 7.4, at 37°C) and different H_2_O_2_ concentrations (0, 2, and 4 mM, at 37°C and pH = 5.5) was measured.

### Photodynamic and photothermal properties

The production of singlet oxygen (^1^O_2_) and hydroxyl radical (·OH) of the wound dressing (PLLA, CN/PLLA, and CN-MnO_2_/PLLA) under 660 nm VL, 808 nm NIR and dual-light irradiation (660 nm VL and 808 nm NIR) were studied using 1,3-diphenylisobenzofuran (DPBF) and methylene blue (MB), respectively. In brief, the dressing was immersed in 1 ml of DPBF (0.6 mg/ml, ethanol) solution and irradiated with 660 nm VL, 808 nm NIR and dual-light irradiation for 30 min. The DPBF solution without dressings was treated under the same conditions and denoted as the blank. H_2_O_2_ (5 μl of 4 mM) was added to the different groups (blank, PLLA, CN/PLLA, CN-MnO_2_/PLLA). After irradiation with VL and/or NIR light for 30 min, 100 μl of DPBF solution was taken out and added into each well in a 96-well plate. The absorption spectra of the DPBF solutions at 360–460 nm was measured by a microplate reader (Varioskan LUX, Thermo Scientific, USA). A decrease in the absorbance peak at 420 nm reflects more ^1^O_2_ production. For the determination of ·OH production, 20 μl of 0.5 M H_2_O_2_ were added to 500 μl of MB (40 mg/l, DI water) solution, and then the dressings (PLLA, CN/PLLA and CN-MnO_2_/PLLA) were added into the MB solution. This was followed by irradiation with different lights (660 nm VL, 808 nm NIR, 660 nm VL + 808 nm NIR dual-light source) for 30 min. MB solution without dressing was treated under the same condition and denoted as the blank. Then, 100 μl of the irradiated solutions were transferred to a 96-well plate, and the absorption spectra of the solutions at 550–750 nm were measured by a microplate reader (Varioskan LUX, Thermo Scientific, USA). A decrease in the absorbance peak at 663 nm reflects more ·OH production by the samples under 660 nm VL, 808 nm NIR and dual-light source irradiation.

The photothermal performance of the wound dressings (PLLA, CN/PLLA, CN-MnO_2_/PLLA) was evaluated by recording their temperature variation after irradiated with 660 nm VL, 808 nm NIR and dual-light irradiation for different times. Briefly, the wound dressing was soaked into 1 ml of PBS in a 1.5 ml EP tube. After irradiation with VL and/or NIR light for different times, the temperature of the soaking solutions was recorded using a temperature curve recorder (EX3008, China). The temperature of PBS without adding dressing under the same irradiation conditions was also determined, which was set as the blank.

### GPx-like activity

GSH consumption of different dressings with or without VL and/or NIR light irradiation was detected using 5,5′ dithiodithio 2-nitrobenzoic acid (DTNB). DTNB can react with reduced GSH to generate yellow 2-nitro-5-thiobenzoic acid (TNB) and oxidized GSH (GSSG), thus showing the GSH consumption ability of different wound dressings under different treatments. Briefly, 25 μl of GSH (3 mg/ml) was added to 270 μl of DMSO (pH = 8.5) solution containing the dressings (PLLA, CN/PLLA, and CN-MnO_2_/PLLA) and incubated for 30 min at 37°C. Afterwards, 5 μl of DTNB (10 mg/ml) was added dropwise into the above solutions to react with the remaining GSH. After 1 min of reaction, the absorbance of the supernatant at 410 nm was measured to detect the consumption of GSH. The GSH consumption without the dressings was set as the blank. GSH consumption can be calculated according to the following formula: loss of GSH (%) = (OD_blank_ − OD_sample_)/OD_blank_ × 100%, where OD_blank_ and OD_sample_ represent the absorbance of the blank and experiential groups, respectively. In addition, the GPx-like activity of the CN-MnO_2_/PLLA dressing with or without VL and/or NIR light irradiation was evaluated by the same method. The influence of VL and/or NIR light and temperature (25 and 42°C) on the GPx-like activity was also investigated using the same procedures.

### Antibacterial performance of the wound dressing

#### Preparation of bacterial medium

*Staphylococcus aureus* (*S. aureus*) and *Pseudomonas aeruginosa* (*P. aeruginosa)* were purchased from American Type Culture Collection (ATCC) and selected as the representative bacteria for antibacterial biofilm tests of the wound dressing. In detail, 3 g of peptone, 3 g of sodium chloride, and 1.2 g of yeast extract powders were dissolved in 300 ml of DI water to prepare the liquid Luria–Bertani (LB) bacterial medium. Subsequently, 4.5 g of agar powder were added into liquid LB medium to obtain solid bacterial medium. The solid bacterial medium was evenly spread on Petri plates. Finally, the liquid and solid LB bacterial medium were sterilized in an autoclave (YXQ-30SII, Shanghai boxun) at 120°C for 30 min.

### Bacterial biofilm disruption

The wound dressing was sterilized via immersion in 75 vol.% ethanol for 2 h and irradiation with ultraviolet (UV) light for 30 min. Then the sterilized dressings were placed into each well in a 96-well plate. Afterwards, 50 μl of bacterial suspension (1 × 10^6^ CFU/ml, CFU, colony forming units) was inoculated in 200 μl of liquid LB bacterial medium, which was seeded on the dressing in the 96-well plate. After coculturing at 37°C for 48 h to form bacterial biofilm, 5 μl of 4 mM H_2_O_2_ was added or not into the different groups and the samples were irradiated with 660 nm VL + 808 nm NIR dual lights for 30 min. Meanwhile, 250 μl of bacterial culture medium (50 μl of bacterial suspension + 200 μl of LB medium) without dressing was used as the blank. To evaluate the biofilm disruption of the dressing, the samples were stained with crystal violet (CV). Briefly, the dressing and bacterial suspensions were removed from the 96-well plate, and then the biofilm in the plate was rinsed with 200 μl of PBS solution and dehydrated with 200 μl of absolute ethyl alcohol for 30 min. Then the absolute ethyl alcohol was taken out of the 96-well plate and 200 μl of the 1.0% CV staining solution was added in the 96-well plate. After natural drying of the CV staining solution, the bacterial biofilms were photographed by a digital camera. For quantitative analysis, the bacterial biofilms in the 96-well plate were dissolved in 100 μl of ethanol solution, then the OD value of the solution at 570 nm was measured using a microplate reader (Varioskan LUX, Thermo Scientific, USA). The antibacterial biofilm rate was calculated using the following formula: antibacterial biofilm rate (%) = (OD_blank_ − OD_sample_)/OD_blank_ × 100%, where OD_blank_ is the absorbance of dissolved natural propagation bacteria biofilm at 570 nm, whereas OD_sample_ represents the absorbance of dissolved bacteria biofilms after different treatments at 570 nm.

### Bacterial biofilm morphology

The morphology of biofilms formed by bacteria on different samples was observed by SEM. In brief, the wound dressings (PLLA, CN/PLLA, and CN-MnO_2_/PLLA) were placed into each well in a 96-well plate. Bacterial suspension (250 μl; 2 × 10^5^ CFU/ml) was inoculated into the dressings. Bacterial culture medium (250 μl; 50 μl of bacterial suspension +200 μl of LB medium) without the dressing was used as the blank. After incubating for 48 h, 5 μl of 4 mM H_2_O_2_ was added or not to the different samples (blank, PLLA, CN/PLLA, and CN-MnO_2_/PLLA) and the samples were irradiated with 660 nm VL + 808 nm NIR dual lights for 30 min. The medium was sucked out and the samples were fixed with 1 ml of glutaraldehyde (2.5%, Macklin) for 30 min. After rinsing with PBS, the samples containing bacteria were dehydrated by concentrated ethanol solution (30, 50, 70, 80, 90, 95, and 100%). Finally, the morphology of the bacterial biofilms on the samples was observed by SEM (EVO18, ZEISS, Germany) at an accelerating voltage of 20 kV.

### Antibacterial effect

The antibacterial effect of different samples was tested by a spread plate experiment. Briefly, the treated bacterial suspensions (2 × 10^5^ CFU/ml) were diluted 10^6^ times, and then 1 μl of each diluted bacterial suspension was removed, dropped onto the agar Petri plates and mixed evenly. After incubating the agar Petri plates at 37°C for 12 h, the bacterial colonies on the plates were photographed by a digital camera and counted using Image J software. The bacterial survival rate was calculated using the following formula: bacterial survival rate (%) = (CFU_blank_ – CFU_sample_)/CFU_blank_ × 100%, where CFU_blank_ denotes the number of bacterial colonies for the groups without 5 μl of 4 mM H_2_O_2_, and CFU_sample_ represents the number of bacterial colonies for the groups with or without 5 μl of 4 mM H_2_O_2_.

### Bacterial membrane permeability

The permeability of bacterial membrane was evaluated using an *o*-Nitrophenyl-β-D-galactopyranoside (ONPG) hydrolysis experiment. Briefly, the bacterial biofilms were incubated on the wound dressing for 48 h with or without the addition of H_2_O_2_ upon exposure to dual-light irradiation. Then the bacterial suspension was diluted with PBS to an OD value of 0.1 at 600 nm. The diluted bacterial suspension (1 ml) was mixed homogeneously with 500 μl of 0.75 M ONPG solution and added dropwise to a 96-well plate. Finally, the absorbance of the mixed solution at 420 nm was measured by a microplate reader (Varioskan LUX, Thermo Scientific).

### Lysozyme sensitivity

The lysozyme sensitivity of *S. aureus* and *P. aeruginosa* was evaluated using lysozyme reagent. Briefly, different wound dressings were placed into each well in a 96-well plate, and then 250 μl of bacterial suspension (2 × 10^5^ CFU/ml) was inoculated on the dressings. After coculturing at 37°C for 48 h, 5 μl of 4 mM H_2_O_2_ was added or not to the different samples. Meanwhile, different groups with/without H_2_O_2_ were irradiated with dual lights for 30 min. Subsequently, 200 μl of the bacterial suspension of the different groups was sucked out and mixed thoroughly with 790 μl of PBS . Finally, 10 μl of lysozyme (1 mg/ml) solution was added to the bacterial suspension and incubated for 1 h at 37°C. Finally, the absorbance of the bacterial suspension in the different groups at 600 nm was measured using a microplate reader (Varioskan LUX, Thermo Scientific).

### Protein leakage

A protein concentration standard curve was prepared based on a BCA protein concentration determination kit (Beyotime, China). The BCA working solution was prepared according to the manufacturer’s instructions. After coculturing at 37°C for 48 h, 5 μl of 4 mM H_2_O_2_ was added or not to the different samples. Meanwhile, the different groups with/without H_2_O_2_ were irradiated with dual lights for 30 min. Afterwards, 20 μl of the treated bacterial solution was pipetted into a 96-well plate and 200 μl of BCA working solution was added. After incubation for 50 min at 37°C, the absorbance of the bacterial solutions of the different groups at 562 nm was measured using a microplate reader (Varioskan LUX, Thermo Scientific).

### Bacterial ROS

The amount of ROS in bacteria with different treatment was detected using a ROS assay kit (2′,7′-Dichlorodihydrofluorescein diacetate, DCFH-DA, Beyotime). Briefly, the treated bacterial solutions were collected in 1.5 ml EP tubes and washed twice with PBS. Afterwards, 200 μl of DCFH-DA (0.01 μM) was added to the bacterial solutions and incubated at 37°C for 30 min under dark conditions. Finally, the green staining images of the different groups (blank, PLLA, CN/PLLA, and CN-MnO_2_/PLLA) were observed using a fluorescence microscope (BX53F2, OLYMPUS, Tokyo, Japan).

### Consumption of GSH in bacteria

The wound dressing was cultured with *S. aureus* and *P. aeruginosa* for 48 h and was then removed. The bacteria in suspension were collected after centrifugation and resuspended with 1 ml of PBS. Subsequently, 15 μl of NaOH solution (0.1 M) and 45 μl of DTNB solution (10 mg/ml) were successively added to the bacterial suspensions. After reacting for 2 min, the absorbance of the reaction solutions at 410 nm was detected using a microplate reader (Varioskan LUX, Thermo Scientific, USA). Meanwhile, the color variation of each group was observed using a digital camera.

### *In vitro* cell assay

The cytocompatibility of the wound dressing was evaluated using L929 cells. Mouse fibroblasts, 2–5 passages, (L929, Procell Life Science&Technology Co., Ltd, Wuhan), were selected to evaluate the cell behaviors of the wound dressing. Briefly, L929 cells were cultured in Dulbecco’s modified Eagle’s medium (DMEM, Invitrogen, USA) with 10% fetal bovine serum (Gibco, USA), 100 μg/ml penicillin and 100 μg/ml streptomycin and incubated at 37°C and 5% CO_2_. The DMEM was replaced every other day. When the confluence of the L929 cells reached 80%, the cells were digested with trypsin–Ethylenediaminetetraacetic acid (EDTA) solution for further cultivation on a large scale.

### Cell cytotoxicity assay

The wound dressing was sterilized via immersion in 75% ethanol solution for 24 h and irradiated with UV light for 12 h. The sterilized dressing (100 mg) was soaked in 1 ml of DMEM at 37°C for 24 h, and then the extracts were collected. The blank was DMEM without the dressing. Cell suspension, 100 μl at a density of 5 × 10^4^ cells/ml, was inoculated into each well of a 96-well plate. After 1 day of incubation, the medium was changed with the extracts of different wound dressings. When the culture time reached the predetermined time points, cell relative viability was measured using a cell counting kit-8 (CCK-8; Solarbio, China). The extracts were removed from the 96-well plate, and then 100 μl of DMEM with 10% CCK-8 working solution was added to each well. After incubation at 37°C for 1 h, the OD value of the solution was measured using a microplate reader (Varioskan LUX, Thermo Scientific, USA) at 450 nm.

### Cell proliferation tests

Cell suspension, 100 μl at a density of 2 × 10^4^ cells/ml, was inoculated into each well of a 96-well plate. After 1 day of incubation, the medium was changed with the extracts of the different wound dressings. After a predetermined time point, the cell number was determined using a CCK-8 kit according manufacturer’s protocol. When the culture time reached Day 3, the cells were stained using calcein-AM (Beyotime, China) and propidum iodide (Beyotime, China) according to the manufacturer’s instructions, and then photographed using a fluorescence microscope (BX53F2, Olympus, Japan).

### Cell healing evaluation

The cell migration of L929 cells after different treatments was evaluated using a scratch-wound assay. L929 cells were cultured in a 6-well plate, and when they reached 90% confluence a thin ‘wound’ was introduced by scratching with a 1 ml pipette tip to simulate a wound microenvironment. The wound dressing was added to the 6-well plate. After irradiation with/without 808 nm NIR light for 30 min, thermal images of the wells were taken using a thermal imaging camera (FLIR ONE PRO, Switzerland). After further incubation for 0 and 24 h, the migration of L929 cells was observed under a light microscope (Primo Vet, Carl Zeiss). The change of wound area with time was calculated using ImageJ software. The wound area (%) = 1 − (*A*_0_ − *A*_t_)/*A*_t_ × 100%, where *A*_0_ represents the wound area at 0 h and *A*_t_ denotes the wound area at predetermined time points.

### *In vivo* experiments

#### Animals and surgical procedures

Sprague–Dawley (SD) male rats (6–8 weeks old, average weight 300 g) were purchased from the Laboratory Animal Science and Technology Center at Jiangxi University of Traditional Chinese Medicine. All the animal experiments complied with the guidelines of Nanchang Medical Experimental Animal Care, and the animal protocols were approved by the Nanchang University Animal Care and Use Committee (protocol number NCULAE-20221031030). All animals were fed and used in accordance with the Animal Management Rules of the Ministry of Health of the People’s Republic of China and the Guidelines for the Care and Use of Laboratory Animals of China.

SD male rats were randomly divided into four groups: the blank, PLLA, CN/PLLA, and CN-MnO_2_/PLLA groups. After general anesthesia with pentobarbital sodium, the anesthetized rats’ backs were shaved and disinfected, and three 8 mm diameter circular wounds were made on the backs. Twenty-five microlitres of the Bacterial suspensions of *S. aureus* (25 μl; 1 × 10^6^ CFU/ml) and *P. aeruginosa* (25 μl;1 × 10^6^ CFU/ml) were removed with a medical syringe and injected into the 8-mm wounds. For this test, rats with purulent symptoms, whose wounds were exposed to natural conditions for 2 days were included. The wound dressings (PLLA, CN/PLLA, and CN-MnO_2_/PLLA) were clamped and sealed with bandages.

### *In vivo* antibacterial test

After irradiation with 660 nm VL + 808 nm NIR for 30 min, the bacteria in the different wounds were extracted and spread on the plate. After culturing for 24 h, the bacterial colonies in the different groups were photographed and counted using ImageJ software (USA). The bacterial survival rate was calculated using the following formula: bacterial survival rate (%) = (CFU_blank_ – CFU_sample_)/CFU_blank_ × 100%, where CFU_blank_ denotes the number of bacterial colonies in the blank groups and CFU_sample_ represents the number of bacterial colonies in the wound dressing groups.

### Wound closure rate

Changes in wound repair at −2, 0, 4, 7 and 14 days were recorded using a digital camera, and the wound closure rate was evaluated using the following formula: wound closure rate (%) = (*A*_−2_ − *A*_d_)/*A*_2_ × 100%. Where *A*_−2_ is the area of wound closure on Day 2 and *A*_d_ is area of wound closure after a specific number of days.

### Histological analysis and immunofluorescence staining

After euthanizing, complete wound tissue samples from each group of rats were collected for histological analysis at postoperative Day 14. The skin tissues were fixed in 4% paraformaldehyde solution and embedded in paraffin wax. Then the skin tissues were sectioned at 4 μm thickness for analysis using a microtome (RM2016, Leica, Germany). Skin tissue sections were stained with hematoxylin and eosin (H&E) and Masson’s trichrome according to the manufacturer’s instructions. In addition, immunofluorescence staining was performed on the skin tissue sections to analyze the expression of inflammatory and vascularization related cytokines. After routine deparaffinization, the sections were treated with an antigen retrieval kit (Servicebio, China) and then boiled in a microwave oven for 30 min. After cooling to room temperature, the sections were washed with PBS on a shaker for destaining purposes. To prevent non-specific primary antibody binding, the sections were placed in 20% goat serum and 0.1% Triton X-100 in PBS for 1 h. Subsequently, the sections were incubated with primary antibodies overnight at 4°C. The primary antibodies for TNF-α, Arg-1, VEGF and BFGF were bought from Wuhan Servicebio Technology Co., Ltd. After applying the primary antibodies, tissue sections were washed three time in TBST, then incubated in dilutions of Alexa 594 goat anti-rabbit secondary antibody (Thermo Fisher Scientific, USA). 4′,6-diamidino-2-phenylindole (DAPI, Servicebio, G1012) was used to counterstain the cell nucleus. Finally, the sections were washed three times for 5 min each in TBST in the dark, and the corresponding immunofluorescence images were obtained using a fluorescence microscope (Olympus, Japan).

### Statistical analysis

Data are presented as the mean ± standard deviation (*n* ≥ 3). The difference between two groups was analyzed using Student’s t-test and Analysis of Variance (ANOVA) with the *post hoc* Scheffe test. Survival curves were compared by the Kaplan–Meier log-rank statistical method. *P* values < 0.05 were considered statistically significant.

## Results

### Characterization of CN and CN-MnO_2_

**Figure 1:**
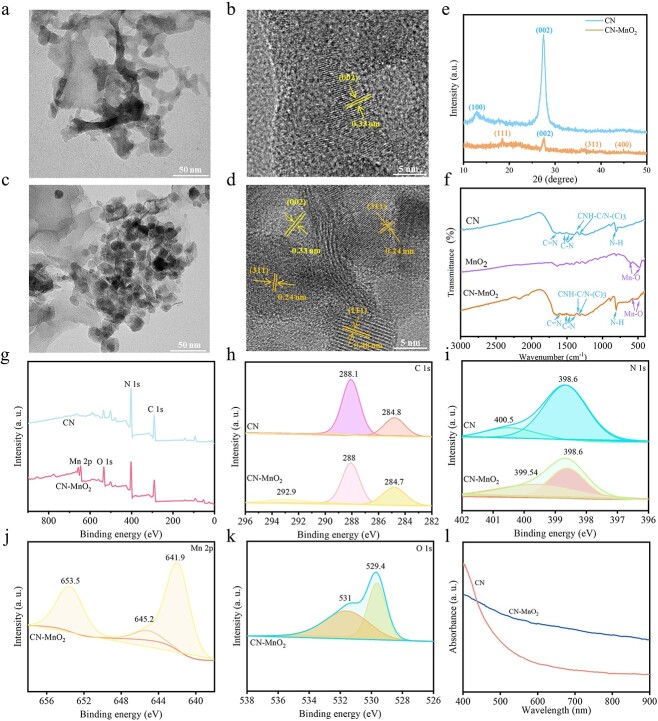
TEM and HR-TEM images of (**a**, **b**) CN and (**c**, **d**) CN-MnO_2_ nanosheets. Scale bars: 50 and 5 nm as indicated. (**e**) XRD pattern of CN and CN-MnO_2_; (**f**) FTIR spectra of CN, MnO_2_, and CN-MnO_2_. (**g**) XPS full spectra of CN and CN-MnO_2_; high-resolution XPS spectra of (**h**) C 1 s, (**i**) N 1 s, (**j**) Mn 2p, and (**k**) O 1 s; and (**l**) UV–Vis–NIR adsorption spectra of CN-MnO_2_ and CN. *TEM* transmission electron microscope, *HR-TEM* high-resolution transmission electron microscope, *MnO_2_* manganese dioxide, *XRD* X-ray Diffraction, *FTIR* Fourier Transform Infrared Spectrometer, *XPS* X-ray photoelectron spectroscopy, *UV-vis-NIR* Ultraviolet-visible spectroscopy-near-infrared, *CN* graphitic phase carbon nitride

The morphology and microstructure of the synthesized CN and CN-MnO_2_ nanocomposites were observed by TEM. As shown in Figure 1a, CN shows a more typical layered structure, displaying a nanosheet-like morphology. The microstructure of the CN nanosheets was further observed by high-resolution TEM (HR-TEM). As shown in Figure 1b, the CN nanosheets have obvious lattice stripes and the corresponding interplanar spacing is 0.33 nm, which corresponds to the (002) crystal plane of g-C_3_N_4_. For the synthesized CN-MnO_2_, many nanoparticles with a size of ~20–30 nm are dispersed evenly on the surface of the CN nanosheets (Figure 1c). After *in situ* growth of MnO_2_ on the surface of the CN nanosheets, the HR-TEM image of CN-MnO_2_ demonstrated that the lattice spacing of 0.33 nm corresponds to the (002) plane of g-C_3_N_4_, and the lattice spacings of 0.48 nm and 0.24 nm are attributed to the (111) and (311) planes of MnO_2_, respectively [16]. In addition, the *in situ* growth of MnO_2_ nanoparticles on the CN nanosheet surface did not destroy the layered structure of the CN nanosheets.

The structures of the atoms and molecules of CN and CN-MnO_2_ were analyzed using XRD. As shown in Figure 1e, there are two obvious characteristic peaks at 12.9 and 27.4° for CN. The wide peak at 12.9° is attributed to the (100) crystal plane, which corresponds to stacking of the 3S-triazine ring structure layer. Another sharp peak at 27.4° belongs to the (002) facet, which is derived from the interlayer stacking of the conjugated aromatic system [17]. Moreover, the diffraction pattern of CN is consistent with that of the g-C_3_N_4_ standard card (JCPDS 87–1526), indicating that the CN nanosheets were synthesized successfully. For the CN-MnO_2_ nanosheets, the diffraction peaks of CN and MnO_2_ are all observed. The diffraction peaks at 19.0 (111), 38.1 (311), and 45.1° (400) are visible in the CN-MnO_2_ diffraction pattern, which matches well with the λ-MnO_2_ standard card (JCPDS 44–0992) [18]. After *in situ* growth of MnO_2_, the g-C_3_N_4_ diffraction peak at 27.4° still appeared, showing that the introduction of MnO_2_ did not affect the crystal structure of g-C_3_N_4_. However, the intensities of the diffraction peaks of g-C_3_N_4_ are significantly reduced, which may be due to the successful growth of MnO_2_*in situ* on CN [19].

The FTIR spectra of CN, MnO_2_, and CN-MnO_2_ are displayed in Figure 1f. The characteristic peak of CN at 1650 cm^−1^ belongs to the stretching vibration of C=N. The typical stretching vibration peaks of C-N appear at 1558, 1456, and 1400 cm^−1^. In addition, the peaks at 1361 and 1322 cm^−1^ are assigned to the stretching vibration of the triazine ring CNH-C or N-(C)_3_. The strong characteristic peak at 808 cm^−1^ is associated with the characteristic breathing-vibration mode of the tri-s-triazine ring. The FTIR MnO_2_ spectrum exhibits absorption peaks at 478 and 597 cm^−1^ corresponding to the stretching vibration of the Mn-O bond [20]. The characteristic peaks of g-C_3_N_4_ and MnO_2_ are visible in the spectra of the CN-MnO_2_ nanosheets, indicating that the *in situ* growth of MnO_2_ basically does not alter the structure of CN [21].

The XPS full spectra indicate that C and N elements are detected in CN, while the synthesized CN-MnO_2_ nanozyme contains C, N, Mn, and O elements (Figure 1g). The high-resolution XPS spectra of C 1 s, N 1 s, Mn 2p, and O 1 s are showed in Figure 1h–k. The C 1 s spectra of CN in Figure 1h show two characteristic peaks centering at 288.1 and 284.8 eV, which correspond to the N-C=N coordination and the sp3-bonded C of C-C or C=C, respectively. However, the high-resolution C 1 s XPS spectra of CN-MnO_2_ have another peak at 292.9 eV, which is assigned to the stretching vibration of the C-O bond. In addition, the C 1 s binding energy of CN-MnO_2_ is slightly lower than that of CN, because the formation of the C-O bond of CN-MnO_2_ leads to the inward shift of C 1 s [22,23]. Figure 1i shows the high-resolution N 1 s spectra of CN and CN-MnO_2_. The dominant peak at 398.6 eV is sp2-hybridized aromatic nitrogen bonded to carbon (C=N-C), and the peak at 400.5 eV is tertiary nitrogen bonded to carbon atoms in the form of N-(C)_3_. The high-resolution Mn 2p XPS spectra are revealed in Figure 1j. Two peaks at 641.9 and 653.5 eV are attributed to Mn 2p3/2 and Mn 2p1/2, respectively. Mn 2p3/2 can be deconvoluted into Mn^2+^, Mn^3+^, and Mn^4+^, respectively. The high-resolution spectra of O 1 s are demonstrated in Figure 1k. There are three subspectral bands of pure MnO_2_. The subspectral bands at 529.4 eV belong to the lattice oxygen (Mn-O-Mn) in MnO_2_, and the weak peaks at 531 eV is the O group of hydroxide ions.

### Physicochemical properties of the dressings

**Figure 2:**
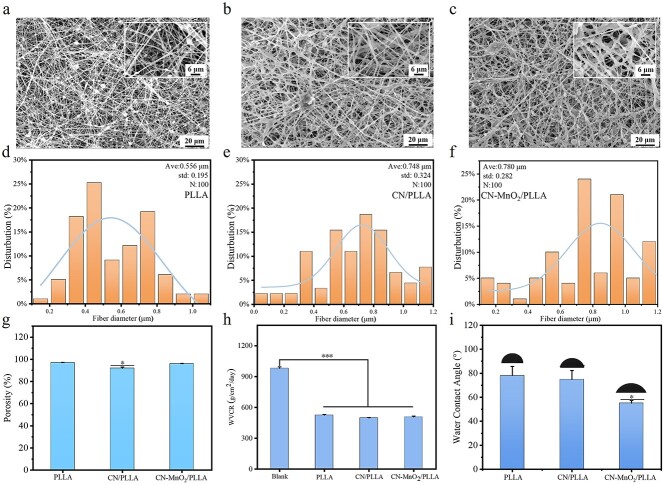
SEM images of the (**a**) PLLA, (**b**) CN/PLLA, and (**c**) CN-MnO_2_/PLLA dressings. Scale bars: 6 and 20 μm as indicated. The fiber diameter distribution of the PLLA (**d**), CN/PLLA (**e**), and CN-MnO_2_/PLLA (**f**) dressings determined from SEM images (*n* = 100). (**g**) Porosity, (**h**) water vapor transmission rate, (**i**) and water contact angle (inserts images is the represent picture of water contact angle) of the different dressings. The significant difference represent ^*^p < 0.05, ^*^^*^^*^p < 0.001 compare with PLLA. *SEM* scanning electron microscopy, *Ave* average, *std* standard deviation, *PLLA* poly-L-lactic acid, *CN* graphitic phase carbon nitride, *MnO_2_* manganese dioxide

The morphology and microstructure of the dressings were observed by SEM. The PLLA dressing exhibits a characteristic beaded-fiber morphology, primarily due to the relatively low concentration of PLLA in the electrostatic spinning solution (Figure 2a) [24]. In the case of the CN/PLLA and CN-MnO_2_/PLLA dressings, it was observed that the particles were evenly distributed in the fibers (Figure 2b, c). The fiber diameter distribution of the PLLA dressing ranges from 200 nm to 1 μm, and the average fiber diameter is ~556 nm. When CN is added into PLLA, the average fiber diameter of the CN/PLLA dressing increases, reaching 748 nm. The average fiber diameter of the CN-MnO_2_/PLLA dressing is further increased to 780 nm.

As shown in Figure 2g, the porosity of all dressings is >90%. Meanwhile, the incorporation of CN and CN-MnO_2_ nanosheets does not obviously influence the porosity of the PLLA dressing. As shown in Figure 2h, the WVTR of the membraneless bottle is 981.2 ± 13.4 g/m^2^/day. The WVTR of the PLLA, CN/PLLA, and CN-MnO_2_/PLLA dressings is 524.9 ± 6.5, 498.4 ± 4.1, and 507.2 ± 9.4 g/m^2^/day, respectively. It was reported that wound dressings with a WVTR between 76 and 9360 g/m^2^/day were able to accelerate wound healing [25].

As shown in Figure 2i, the WCA of PLLA dressing is 78°, displaying obvious hydrophobicity. The incorporation of CN slightly reduces the hydrophobicity of the PLLA dressing, and the WCA of the CN/PLLA dressing is 74°. By contrast, the WCA of the CN-MnO_2_/PLLA dressing is 55°, indicating that the incorporation of CN-MnO_2_ significantly enhances the hydrophilicity of the PLLA dressing.

### Peroxidase activity

**Figure 3:**
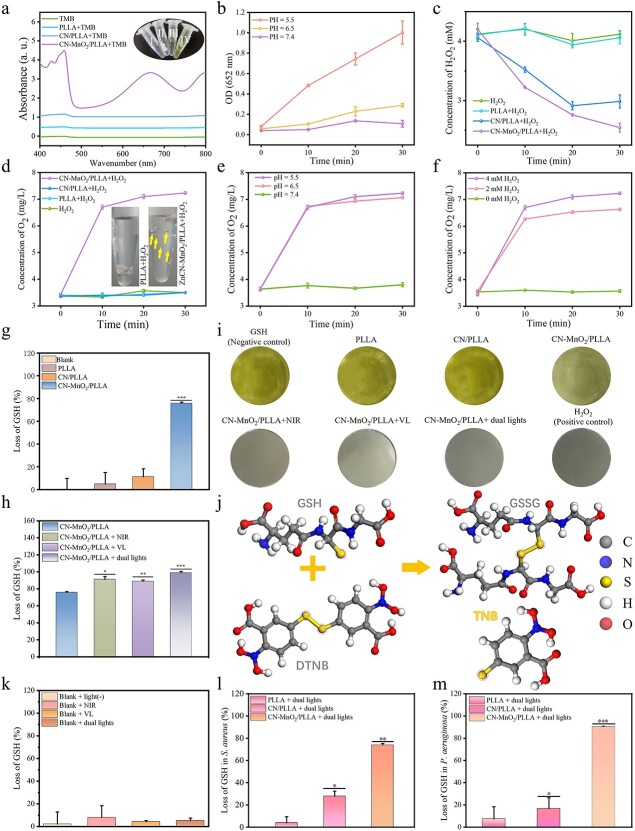
*In vitro* evaluation on the H_2_O_2_ and GSH consumption. (**a**) UV–Vis absorption spectra of TMB solutions treated with different groups (TMB, PLLA+TMB, CN/PLLA+TMB, CN-MnO_2_/PLLA+TMB). Color variation images (insert) of TMB solutions without treatment (I) and treated with the PLLA (II), CN/PLLA (III), and CN-MnO_2_/PLLA (IV). (**b**) Peroxidase activity of the CN-MnO_2_/PLLA dressing under different pH conditions (pH = 5.5, 6.5 or 7.4). (**c**) Changes of H_2_O_2_ concentration in the solutions treated with different groups (H_2_O_2_, PLLA+ H_2_O_2_, CN/PLLA+ H_2_O_2_, CN-MnO_2_/PLLA+ H_2_O_2_) over time at pH 5.5. (**d**) Concentration of O_2_ generated in different groups (H_2_O_2_, PLLA+ H_2_O_2_, CN/PLLA+ H_2_O_2_, CN-MnO_2_/PLLA+ H_2_O_2_), and O_2_ bubble images in the PLLA and CN-MnO_2_/PLLA groups (insert). (**e**) Concentration of O_2_ generated from CN-MnO_2_/PLLA at different pHs (5.5, 6.5 and 7.4). (**f**) Concentration of O_2_ generated from CN-MnO_2_/PLLA in different amounts of H_2_O_2_ (0, 2, and 4 mM). (**g**) Loss of GSH with blank, PLLA, CN/PLLA, and CN-MnO_2_/PLLA with no light. (**h**) Loss of GSH by CN-MnO_2_/PLLA under different light (blank, NIR, VL, dual lights) irradiation. (**i**) Images of the color reaction of DTNB with GSH in pore. (**j**) Mechanism of color reaction of DTNB with GSH. (**k**) Loss of GSH in blank after different treatments (blank, NIR, VL, dual lights). Loss of GSH in the bacteria *S. aureus* (**l**) and *P. aeruginosa* (**m**) grown on different groups (PLLA, CN/PLLA, CN-MnO_2_/PLLA) without H_2_O_2_ for 30 min under dual-light (660 nm VL + 808 nm NIR) irradiation. The significant difference represent ^*^p < 0.05, ^*^^*^p < 0.01, ^*^^*^^*^p < 0.001 compare with PLLA. *UV-Vis* Ultraviolet-visible spectroscopy, *TMB* 3,3′,5,5′-Tetramethylbenzidine, *PLLA* poly-L-lactic acid, CN graphitic phase carbon nitride, *MnO_2_* manganese dioxide, *pH* pondus hydrogenii, *H_2_O_2_* hydrogen peroxide, *VL* visible light, *NIR* near-infrared, *GSH* glutathione, GSSG oxidized glutathione, *DTNB* 5,5′ dithiodithio 2-nitrobenzoic acid, *TNB* 2-nitro-5-thiobenzoic acid, *O_2_* oxygen

The peroxidase activity of the different dressings is evaluated using TMB. The ultraviolet–visible spectroscopy (UV–Vis) absorption spectra and color variation of TMB solutions treated with different samples are shown in Figure 3a. When the PLLA dressing is immersed in an acetic acid solution containing TMB, the TMB solution shows no color change, indicating that PLLA does not possess peroxidase activity. In the CN/PLLA group, the TMB solution showed a weak yellow color, along with a fine absorption peak at 450 nm. This indicated that the CN/PLLA dressing has weak peroxidase activity. Meanwhile, the color of the TMB solution for the CN-MnO_2_/PLLA group changes from colorless to yellow. Meanwhile, an obvious absorption peak at 450 nm is observed in the UV–Vis absorption spectrum of the CN-MnO_2_/PLLA group, demonstrating that the CN-MnO_2_/PLLA sample has strong peroxidase activity.

Due to the significant pH-dependence of peroxidase activity on H_2_O_2_ consumption, the CN-MnO_2_/PLLA dressing is immersed in PBS with different pH values (pH = 7.4, 6.5, or 5.5) to test its peroxidase activity. Figure 3b shows that the CN-MnO_2_/PLLA does not show a significant increase in OD value at 652 nm with increasing immersion time when the pH of PBS is 7.4 and 6.5, indicating weak peroxidase activity in neutral and weak acidic environments. Since the pH range within the biofilm is 4.5–6.5, the peroxidase activity of the CN-MnO_2_/PLLA was further examined at pH = 5.5 [4]. It was found that the OD value at 652 nm of CN-MnO_2_/PLLA increases gradually over time when immersed in PBS with a pH of 5.5, showing the excellent peroxidase activity of CN-MnO_2_/PLLA in an acidic environment.

The color development reaction between TiSO_4_ and H_2_O_2_ was measured at pH = 5.5 to assess the consumption of H_2_O_2_ by the different groups. As shown in Figure 3c, there is almost no consumption of H_2_O_2_ in the blank and PLLA groups with the extension of time. However, the concentration of H_2_O_2_ for the CN/PLLA and CN-MnO_2_/PLLA groups decreases rapidly with the prolongation of immersion time, suggesting significant ability to consume H_2_O_2_.

### Catalase activity

The hypoxic within biofilms limits the efficiency of PDT of CN, MnO_2_ possess good catalase activity. The concentration of O_2_ generated from the different samples was evaluated. As shown in Figure 3d, it is evident that the concentration of O_2_ in the PLLA and CN/PLLA groups hardly changes with extension of the immersion time. By contrast, the concentration of O_2_ in the solution gradually increases over time. When the immersion time is 30 min, the concentration of O_2_ in the CN-MnO_2_/PLLA group reaches 7.3 mg/l. It is clear that many O_2_ bubbles exist in the solution containing the CN-MnO_2_/PLLA dressing, while almost no bubbles were visible in the PLLA group. These results showed that the CN-MnO_2_/PLLA dressing was able to catalyze H_2_O_2_ to produce O_2_, indicating good catalase activity.

In addition, the catalase activity of the CN-MnO_2_/PLLA dressing at different H_2_O_2_ concentrations and pH values was further investigated. As shown in Figure 3e, there is no generation of O_2_ when the concentration of H_2_O_2_ is 0 mM. Meanwhile, the O_2_ generation effect of the CN-MnO_2_/PLLA sample shows significant differences at H_2_O_2_ concentrations of 2 and 4 mM. The concentration of O_2_ in the solution with 4 mM H_2_O_2_ is evidently high than that with 2 mM H_2_O_2_ at any time. In addition, the concentration of O_2_ in the solution is gradually enhanced with increasing immersion time. The influence of pH on the catalase activity of the CN-MnO_2_/PLLA dressing was also explored. As exhibited in Figure 3f, the CN-MnO_2_/PLLA dressing possesses high catalase activity under acidic conditions (pH = 5.5, 6.5). In contrast, when the pH value is adjusted to 7.4, almost no O_2_ is produced.

### GPx activity

As shown in Figure 3g, the consumption of GSH in the PLLA and CN/PLLA groups is negligible after 30 min of immersion under dark conditions. By contrast, the CN-MnO_2_/PLLA dressing has the highest consumption of GSH, with a GSH loss of >70%, displaying excellent GPx activity. The consumption of GSH by the CN-MnO_2_/PLLA dressing was further investigated under light irradiation conditions. As shown in Figure 3h, the GSH consumption is further increased by NIR and/or VL irradiation. Moreover, the GSH loss by CN-MnO_2_/PLLA reaches 99% under dual-light irradiation (660 nm + 808 nm). The probable reason is that the mild photothermal effect of CN-MnO_2_/PLLA under NIR irradiation further enhances the GPx activity [26]. In addition, ROS produced from the dressing by irradiating VL can also consume GSH. In Figure 3i, the color of the solutions for the PLLA and CN/PLLA groups showed minimal difference compared to the blank, displaying a deep yellow color. By adding CN-MnO_2_/PLLA to the GSH solution, the yellow color of the solution is significantly lightened. The color variation of the solutions containing the CN-MnO_2_/PLLA dressing with different light source illumination was also studied. It is evident that the color of the solutions gradually become lighter when exposed to different light sources (Figure 3i). Furthermore, the solution turns completely colorless under dual-light irradiation, indicating a higher depletion of GSH in the solution.

The illustration in Figure 3j depicts the reaction mechanism of color development between DTNB and GSH. It is known that DTNB reacts with the free sulfhydryl group of GSH to produce a mixed disulfide and TNB. In this chemical reaction, the DTNB specifically targets the coupling group of a free sulfhydryl group (R-S-): GSH + DTNB → GSSG + TNB. The remaining GSH in the solution then oxidizes DTNB, resulting in the formation of yellow TNB. The colorimetric reaction between DTNB and GSH further confirms that the CN-MnO_2_/PLLA dressing exhibits excellent GPx activity and consumes more GSH under laser light irradiation. Furthermore, to determine if the consumption of GSH is caused by light source irradiation, the effect of light irradiation on the loss of GSH was conducted. As shown in Figure 3k, the consumption of GSH does not significantly increase, indicating that both the 660 nm VL light and 808 nm NIR light sources did not noticeably decrease GSH levels. We further analyzed the consumption of GSH within the bacteria by utilizing DTNB (Figure 3l, m). It has been shown that the CN/PLLA dressing consumes a portion of GSH due to the production of ROS when exposed to dual-light irradiation [27]. Additionally, the loss of GSH by the CN-MnO_2_/PLLA dressing reached 73% in *S. aureus* and 90% in *P. aeruginosa* due to the excellent GPx activity of MnO_2_.

**Figure 4:**
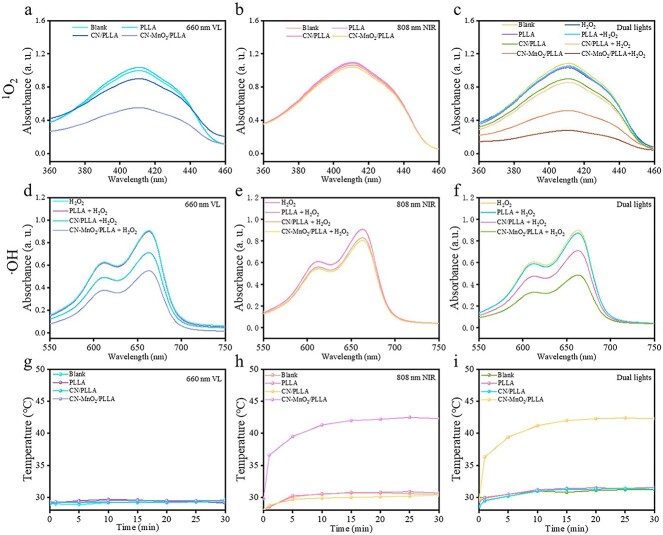
^1^O_2_ generation in different samples (blank, PLLA, CN/PLLA and CN-MnO_2_/PLLA) under (**a**) 660 nm VL, (**b**) 808 nm NIR, (**c**) dual-light (660 nm VL + 808 nm NIR) irradiation. ·OH generation in different samples under (**d**) 660 nm VL, (**e**) 808 nm NIR, and (**f**) and dual-light irradiation. Photothermal warming curve of different samples under (**g**) 660 nm VL, (**h**) 808 nm NIR, and (**i**) dual-light irradiation. *^1^O*_*2*_ singlet oxygen, *PLLA* poly-L-lactic acid, *CN* graphitic phase carbon nitride, *MnO*_2_ manganese dioxide, *VL* visible light, *NIR* near-infrared, *⋅OH* hydroxyl radical

### Photodynamic and photothermal properties

The photodynamic properties of different dressings under VL and/or NIR irradiation were evaluated using DPBF and MB. DPBF and MB are used to detect the production of singlet oxygen (^1^O_2_) and hydroxyl radical (·OH), respectively. The absorbance of the solution at 410 nm in the PLLA group did not change obviously under 660 nm VL irradiation in comparison to that of the blank (Figure 4a). However, the absorbance of the solution in both the CN/PLLA and CN-MnO_2_/PLLA groups tended to decrease, and the absorbance of the CN-MnO_2_/PLLA group was the lowest. These experimental results illustrate that the CN/PLLA and CN-MnO_2_/PLLA dressings are able to produce ^1^O_2_ after 660 nm VL irradiation, and the CN-MnO_2_/PLLA sample yields the greatest quantity of ^1^O_2_. However, the absorbance of the different groups at 410 nm was not obviously altered upon exposure to 808 nm NIR light, indicating that the dressings did not generate ^1^O_2_ under irradiation with an 808 nm NIR light source (Figure 4b).

The CN-MnO_2_/PLLA dressing can decompose H_2_O_2_ into O_2_ due to the catalase activity of MnO_2_. The generated O_2_ effectively alleviated the hypoxia caused by the bacterial biofilm and provided sufficient O_2_ for the production of ^1^O_2_. Therefore, the absorbance of DPBF solutions with or without H_2_O_2_ in different groups under dual-light (660 nm VL and 808 nm NIR) irradiation was investigated. As exhibited in Figure 4c, there was no noticeable difference in the absorbance between the blank and PLLA groups after the addition of the H_2_O_2_ solution. In contrast, after adding H_2_O_2_ solution to the CN/PLLA or CN-MnO_2_/PLLA groups, the absorbance of the DPBF solution at 410 nm decreased significantly in contrast to that without H_2_O_2_, indicating that more ^1^O_2_ was produced in the presence of H_2_O_2_. The above results demonstrate that the dressing produces ^1^O_2_ only under 660 nm VL irradiation and does not produce ^1^O_2_ under 808 nm NIR light activation [28]. In addition, more ^1^O_2_ for the CN/PLLA and CN-MnO_2_/PLLA dressings can be produced in a H_2_O_2_ environment after VL irradiation.

MB can be used to detect the amount of ·OH. The absorbance of the MB solution at 662 nm decreases when it reacts with ·OH. As shown in Figure 4d, the absorbance of the PLLA group did not significantly change compared with that of the blank group under 660 nm VL irradiation, while the absorbance intensity at 662 nm corresponding to CN/PLLA and CN-MnO_2_/PLLA clearly decreased. Moreover, the CN-MnO_2_/PLLA group had the lowest OD value at 662 nm. These results revealed that the CN/PLLA and CN-MnO_2_/PLLA dressings can consume H_2_O_2_ to produce ·OH under 660 nm laser excitation. The CN-MnO_2_/PLLA dressing produces the most ·OH, further indicating that CN-MnO_2_/PLLA has the strongest PDT efficiency. In addition, the absorbance of the MB solution at 550–750 nm did not obviously change upon irradiation with 808 nm NIR light. Then, the absorbance of the CN/PLLA and CN-MnO_2_/PLLA groups slightly decreased at 662 nm, indicating that no obvious ·OH was generated upon excitation with 808 nm light (Figure 4e). As shown in Figure 4f, the variation trend of the absorbance of the MB solution with different treatments under dual-light irradiation (660 nm VL + 808 nm NIR) was the same as that under 660 nm VL irradiation. The probable reason for the good photodynamic effect of the CN-MnO_2_/PLLA dressing is that the *in situ* growth of MnO_2_ on the CN surface facilitates electron–hole transport and promotes more ROS generation [17].

The temperature variation curves of the different dressings after immersion in PBS under irradiation with VL and/or NIR light for different durations are displayed in Figure 4g–i. There was no significant temperature change in the blank, PLLA, and CN/PLLA groups under 660 nm VL single-light irradiation. In contrast, under 808 nm NIR light irradiation, the CN-MnO_2_/PLLA dressing obviously increased the temperature. When the irradiation time was 30 min, the temperature of the immersion solution with the CN-MnO_2_/PLLA dressing reached 42°C. Upon exposure to 660-nm VL **+** 808-nm NIR dual-light irradiation, the temperature changes in the different groups were the same as those observed under 808-nm NIR single-light irradiation. From the above experimental results, it can be concluded that the CN-MnO_2_/PLLA dressing produces mild photothermal effects only under 808 nm laser stimulation [29].

### Bacterial biofilm evaluation

**Figure 5:**
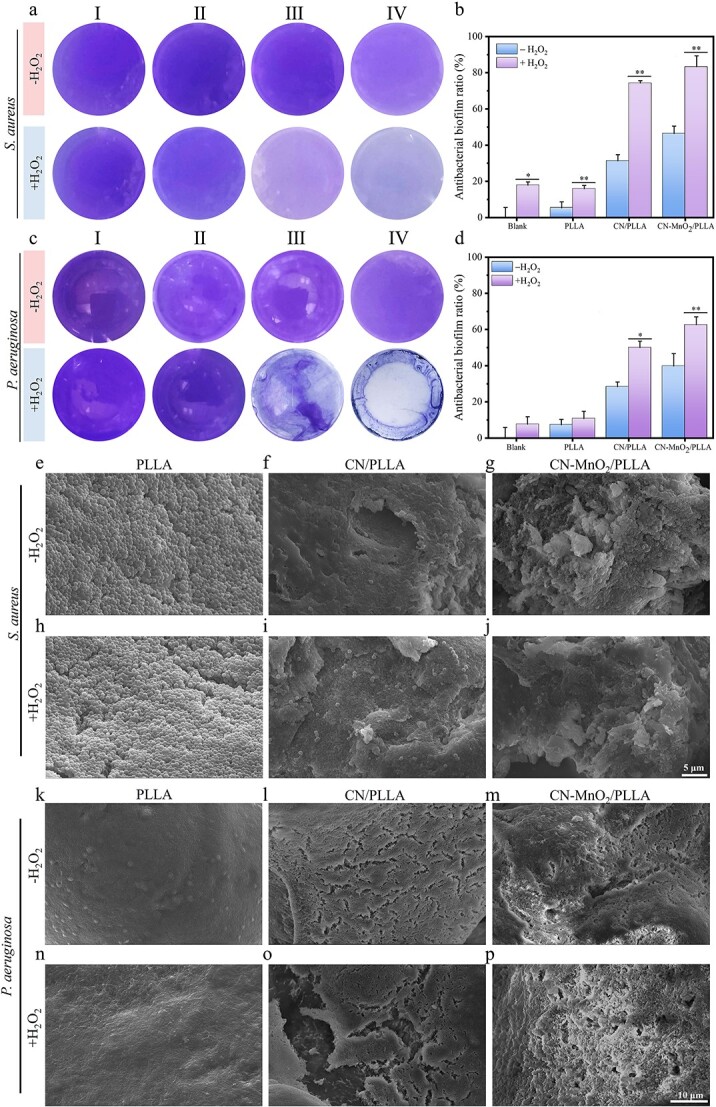
Representative biofilm staining images of (**a**) *S. aureus* and (**c**) *P. aeruginosa* with or without H_2_O_2_ for 30 min under dual-light (660 nm VL + 808 nm NIR) irradiation. The corresponding antibacterial biofilm ratio of (**b**) *S. Aureus* and (**d**) *P. Aeruginosa*. I, II, III, and IV represent the four groups of blank, PLLA, CN/PLLA, and CN-MnO_2_/PLLA, respectively. Images of biofilm morphology of *S. aureus* on the PLLA (**e**, **h**), CN/PLLA (**f**, **i**), and CN-MnO_2_/PLLA (**g**, **j**) with or without H_2_O_2_. Scale bar: 5 μm. Images of biofilm morphology of *P. aeruginosa* on the PLLA (**k**, **n**), CN/PLLA (**l**, **o**), and CN-MnO_2_/PLLA (**m**, **p**) with or without H_2_O_2_. The significant difference represent ^*^p < 0.05, ^*^^*^p < 0.01 compare with without H_2_O_2_. Scale bar: 10 μm. *S. aureus staphylococcus aureus*, *P. aeruginosa pseudomonas aeruginosa*, *H_2_O_2_* hydrogen peroxide, *VL* visible light, *NIR* near-infrared, *PLLA* poly-L-lactic acid, *CN* graphitic phase carbon nitride, *MnO_2_* manganese dioxide

Generally, excessive H_2_O_2_ is present in infected wounds [30,31]. Therefore, to mimic the microenvironment, 5 μl of 4 mM H_2_O_2_ was added when evaluating antibacterial effects. After 48 h of bacterial biofilm culture, the biofilms were exposed to dual-light irradiation for 30 min in the presence or absence of H_2_O_2_. As shown in Figure 5a and b, H_2_O_2_ itself has the ability to partially eliminate *S. aureus* bacterial biofilms. Moreover, the biofilm elimination ability of the CN/PLLA sample under dual-light irradiation was significantly enhanced when H_2_O_2_ was added, resulting in an antibacterial biofilm percentage of 74%. The probable reason is that H_2_O_2_ can be converted into ·OH by peroxidase, which supplies abundant ROS [32]. When dual lights are applied to the CN-MnO_2_/PLLA sample, there is a further increase in bacterial biofilm destruction. After the addition of H_2_O_2_, the antibacterial biofilm ratio of *S. aureus* for the CN-MnO_2_/PLLA group reached 83%. In Figure 5c and d, the biofilms in different groups (blank, PLLA, CN/PLLA, and CN-MnO_2_/PLLA) showed various degrees of disruption after dual-light irradiation for 30 min in the presence or absence of H_2_O_2_. Similarly, the biofilms of both the blank and PLLA groups showed different degrees of damage in the presence or absence of H_2_O_2_. The disruption of *P. aeruginosa* bacterial biofilms in the CN/PLLA group upon irradiation with dual lights in the presence of H_2_O_2_ reached >50%. The antibacterial biofilm ratio (62%) of the CN-MnO_2_/PLLA sample was further enhanced.

**Figure 6:**
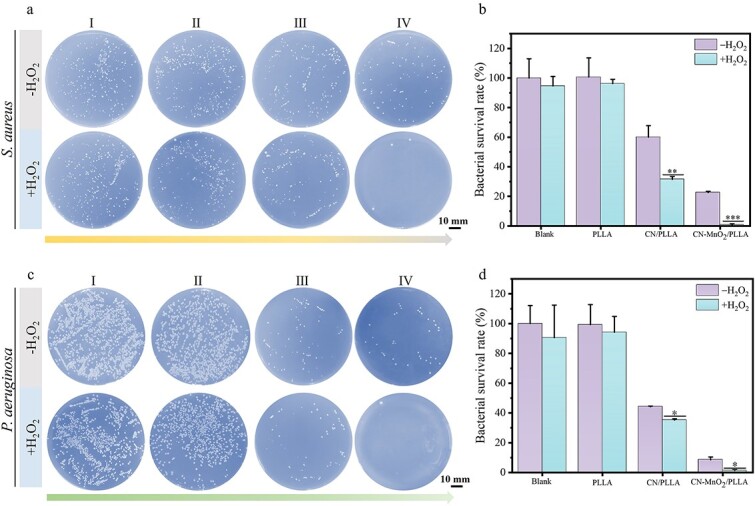
(**a**) Images and (**b**) the corresponding bacterial survival rate of *S. aureus* colonies in the (I) blank, (II) PLLA, (III) CN/PLLA, and (IV) CN-MnO_2_/PLLA groups with or without H_2_O_2_ for 30 min under dual-light (660 nm VL + 808 nm NIR) irradiation. (**c**) Images and (**d**) the corresponding bacterial survival rate of *P. aeruginosa* colonies in the (I) blank, (II) PLLA, (III) CN/PLLA and (IV) CN-MnO_2_/PLLA groups with or without H_2_O_2_ for 30 min under dual-light (660 nm VL + 808 nm NIR) irradiation. Scale bar: 10 mm. The significant difference represent ^*^p < 0.05, ^*^^*^p < 0.01, ^*^^*^^*^p < 0.001 compare with without H_2_O_2_. *S. aureus staphylococcus aureus*, *P. aeruginosa pseudomonas aeruginosa*, *PLLA* poly-L-lactic acid, *CN* graphitic phase carbon nitride, *MnO_2_* manganese dioxide, *VL* visible light, *NIR* near-infrared, *H_2_O_2_* hydrogen peroxide

The surface morphology, internal structure, and density of the bacterial biofilms were observed via SEM. As shown in Figure 5e and h, the *S. aureus* bacterial biofilms on the PLLA dressing with or without H_2_O_2_ grew well and exhibited a tight arrangement. Moreover, the surface of *S. aureus* was smooth and did not show obvious folds, indicating that there was no obvious destruction of the dense biofilms on the PLLA dressing. For the CN/PLLA dressing with or without H_2_O_2_, the *S. aureus* biofilms displayed noticeable folds and rupture (Figure 5f and i). In the case of CN-MnO_2_/PLLA, more prominent folds and destruction are observed (Figure 5g and j). The morphology of the *P. aeruginosa* bacterial biofilm on the dressings with different treatments is shown in Figure 5k–p. It is evident that the biofilm of *P. aeruginosa* appears to be denser than the *S. aureus* biofilm. Regardless of whether H_2_O_2_ is added, the biofilms on the PLLA dressing cannot be destroyed by dual-light irradiation. In contrast, the biofilms on CN/PLLA and CN-MnO_2_/PLLA without H_2_O_2_ exhibited many cracks, suggesting disruption of the biofilm structure (Figure 5l and m). Furthermore, the addition of H_2_O_2_ led to further destruction of the biofilms on both the CN/PLLA and CN-MnO_2_/PLLA dressings (Figure 5o and p).

### Antibacterial property

The spread plate results of *S. aureus* and *P. aeruginosa* with different treatments are displayed in Figure 6. There is no noteworthy disparity in the quantity of bacterial colonies observed between the PLLA and the blank groups when exposed to identical conditions (Figure 6a). This suggests that the presence of H_2_O_2_ and dual-light irradiation does not exert a substantial influence on the bacterial survival rate. However, the number of bacterial colonies significantly decreases for the CN/PLLA group with or without H_2_O_2_ when subjected to dual-light irradiation as compared to both the blank and PLLA groups (Figure 6a and b). Moreover, the addition of H_2_O_2_ in the CN/PLLA group further enhances the antibacterial effect after dual-light irradiation. The bacterial survival rate of *S. aureus* decreases to 30%. In contrast, the survival rate of *S. aureus* for the CN-MnO_2_/PLLA group with dual-light irradiation is the lowest. Interestingly, almost no bacterial colony is observed after the addition of H_2_O_2_ into the CN-MnO_2_/PLLA group. The survival rate of *P. aeruginosa* on the dressings with different treatments shows similar trends (Figure 6c and d). The survival rate of *P. aeruginosa* on the CN/PLLA dressing with H_2_O_2_ is 35.7% under dual-light irradiation. However, the bacterial survival rate for the CN-MnO_2_/PLLA group without H_2_O_2_ is <10%, The addition of H_2_O_2_ significantly reduces the bacterial survival rate to only 2% for the CN-MnO_2_/PLLA group under dual-light irradiation.

### Antibacterial mechanism

**Figure 7:**
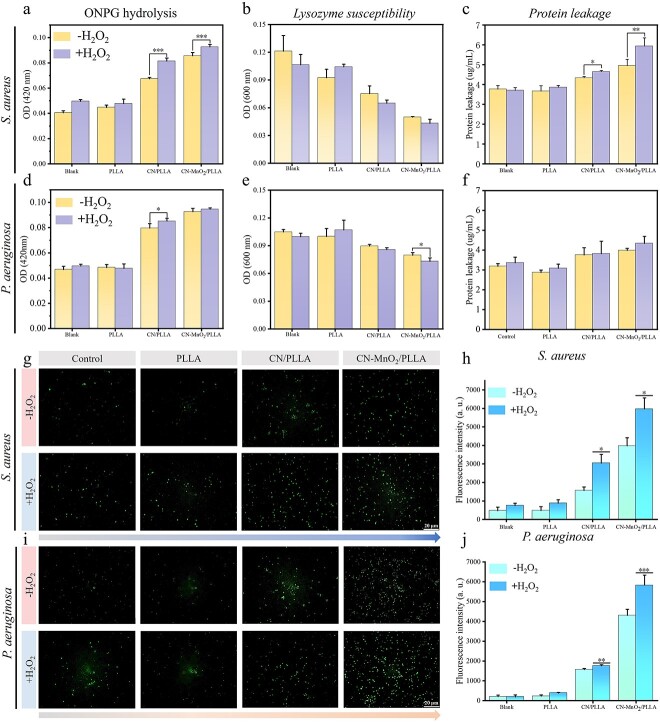
Permeability of bacterial membrane of (**a**) *S. aureus* and (**d**) *P. aeruginosa* in different groups with or without H_2_O_2_ under dual-light (VL + NIR) irradiation. Lysozyme susceptibility of (**b**) *S. aureus* and (**e**) *P. Aeruginosa* in different groups with or without H_2_O_2_ under dual-light irradiation. Protein leakage of (**c**) *S. aureus* and (**f**) *P. aeruginosa* in different groups with or without H_2_O_2_ under dual-light irradiation. ROS fluorescence images of (**g**) *S. aureus* and (**i**) *P. aeruginosa* in the blank, PLLA, CN/PLLA, and CN-MnO_2_/PLLA groups with or without H_2_O_2_ under dual-light (660 nm VL + 808 nm NIR) irradiation for 30 min. Scale bar: 20 μm. The corresponding fluorescence intensity of (**h**) *S. aureus* and (**j**) *P. aeruginosa* in different groups with or without H_2_O_2_ for 30 min under dual-light irradiation. The significant difference represent ^*^p < 0.05, ^*^^*^p < 0.01, ^*^^*^^*^p < 0.001 compare with without H_2_O_2_. *S. aureus staphylococcus aureus*, *P. aeruginosa pseudomonas aeruginosa*, *ONPG* O-Nitrophenyl-b-D-galactopyranoside, *VL* visible light, *NIR* near-infrared, *H_2_O_2_* hydrogen peroxide, *OD* optical density, *ROS* reactive oxygen, *PLLA* poly-L-lactic acid, C*N* graphitic phase carbon nitride, *MnO_2_* manganese dioxide

The permeability of the bacterial membrane was also evaluated using ONPG. β-Galactosidase in bacteria is capable of hydrolyzing ONPG, resulting in the formation of *o*-nitrophenol, which is a yellow product. When the permeability of the bacterial membrane changes, ONPG can easily penetrate the membrane and enter the bacteria. As a result, the rate of enzymatic hydrolysis increases, leading to the production of a larger quantity of yellow products. Therefore, the assessment of bacterial membrane permeability can be performed by quantifying the number of yellow products. The results of the bacterial membrane permeability of *S. aureus* and *P. aeruginosa* are displayed in Figure 7a and d. The permeability of the bacterial membrane in the PLLA group with or without H_2_O_2_ did not increase compared to that in the blank group. After adding H_2_O_2_, there was no significant increase in bacterial membrane permeability in either the blank or PLLA groups. The permeability of the bacterial membrane in the CN/PLLA and CN-MnO_2_/PLLA groups significantly increased compared to that in the PLLA and blank groups. Furthermore, the introduction of H_2_O_2_ into both the CN/PLLA and CN-MnO_2_/PLLA groups can further enhance the permeability of the bacterial membrane. Among these groups, the CN-MnO_2_/PLLA group with the addition of H_2_O_2_ exhibited the strongest enhancement effect on bacterial membrane permeability. The primary reason is that Mn^4+^ released from the CN-MnO_2_/PLLA dressing can serve as a catalyst for H_2_O_2_, leading to the production of O_2_ [33]. O_2_ serves as the raw material for the photodynamic reaction of CN when exposed to VL, thus generating more ROS. MnO_2_ also possesses a mild photothermal effect; when combined with the photodynamic effect, MnO_2_ can synergistically increase bacterial membrane permeability.

To further investigate the impact of the dressings on the bacterial membranes, the susceptibility of the various groups to lysozyme was evaluated. As shown in Figure 7b and e, the lysozyme susceptibility of the PLLA group with or without H_2_O_2_ was not obviously different from that of the blank group. The CN/PLLA group possessed greater lysozyme susceptibility than the PLLA and blank groups. Bacteria cultured on the CN-MnO_2_/PLLA dressing were the most sensitive to lysozyme. Furthermore, the addition of H_2_O_2_ to the CN/PLLA and CN-MnO_2_/PLLA groups further enhanced the lysozyme susceptibility. In general, disruption of the bacterial membrane is accompanied by protein leakage within the bacteria. A BCA protein quantification kit was used to detect protein leakage after bacterial membrane disruption. As shown in Figure 7c and f, the protein leakage in the PLLA group, regardless of the presence of H_2_O_2_, did not significantly increase compared to that in the blank group. In contrast, the CN/PLLA and CN-MnO_2_/PLLA groups clearly demonstrated elevated protein leakage compared to the PLLA and blank groups. Moreover, the introduction of H_2_O_2_ into the CN/PLLA and CN-MnO_2_/PLLA groups further enhanced the protein level of the bacteria. Additionally, it is worth mentioning that the protein leakage level in the CN-MnO_2_/PLLA group with H_2_O_2_ was the highest among all groups.

The ROS level in the bacteria with different treatments was detected by DCFH-DA. As displayed in Figure 7g and i, a small number of green fluorescent dots are present in both the blank and PLLA groups, regardless of the presence of H_2_O_2_. This can be attributed to the production of ROS through aerobic respiration [34,35]. The green fluorescence observed in the CN/PLLA group is noticeably higher compared to the blank. Similarly, the addition of H_2_O_2_ to the CN/PLLA group leads to the decomposition and release of O_2_, significantly increasing the amount of ROS. Therefore, the green fluorescence level in the CN/PLLA group with H_2_O_2_ is stronger than that without H_2_O_2_ (Figure 7h and j). Interestingly, the CN-MnO_2_/PLLA group exhibits a noteworthy enhancement in green fluorescence intensity when exposed to dual-light irradiation compared with other groups. When H_2_O_2_ is added, the intensity of green fluorescence in the CN-MnO_2_/PLLA group is further enhanced. The corresponding green fluorescence intensity was consistent with the semi-quantitative results. The above results offer further evidence supporting the involvement of multiple factors in the death process of bacteria cultivated on the dressings.

### Cytotoxicity and cell migration

**Figure 8:**
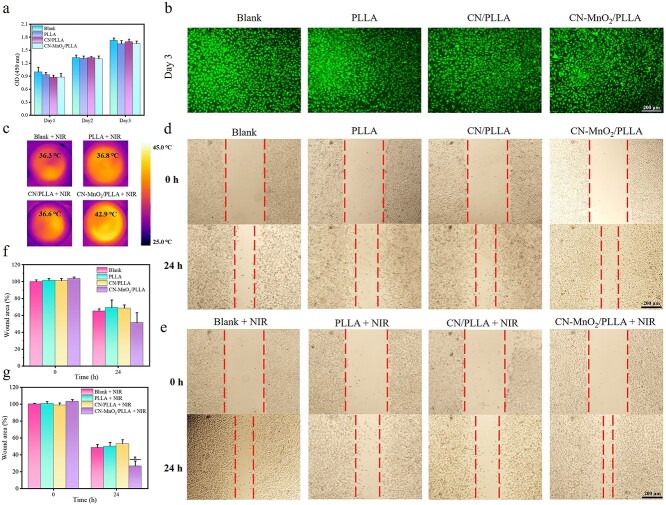
Cytotoxicity and cell migration evaluation. (**a**) Proliferation of L929 cells co-cultured with different groups (blank, PLLA, CN/PLLA, and CN-MnO_2_/PLLA) for 1, 2, and 3 days. (**b**) Live/dead fluorescence staining images of L929 cells co-cultured with different groups (blank, PLLA, CN/PLLA, and CN-MnO_2_/PLLA) at 3 days. Scale bar: 200 μm. (**c**) Infrared thermal images of L929 cells in different groups (blank, PLLA, CN/PLLA, and CN-MnO_2_/PLLA) after irradiation with NIR light for 30 min. Scale bar: 700 mm. Optical images of the wound healing of L929 cells (**d**) without or (**e**) with NIR light irradiation after incubation with different groups (blank, PLLA, CN/PLLA, and CN-MnO_2_/PLLA) for 0 and 24 h. Scale bar: 200 μm. The relative wound area of L929 cells (**f**) without or (**g**) with NIR light irradiation after incubation with different groups (blank, PLLA, CN/PLLA, and CN-MnO_2_/PLLA) for 0 and 24 h. The significant difference represent ^*^p < 0.05 compare with Blank+NIR at 24 h. *NIR* near-infrared, *OD* optical density, *PLLA* poly-L-lactic acid, *CN* graphitic phase carbon nitride, *MnO_2_* manganese dioxide

PLLA is chosen as the matrix for wound dressing due to its good biocompatibility, biodegradability, and mechanical properties [36]. However, the introduction of CN and CN-MnO_2_ may affect the biological properties. Therefore, the cytocompatibility of the different dressings (blank, PLLA, CN/PLLA, and CN-MnO_2_/PLLA) was assessed using live/dead staining and CCK-8 kits. As show in Figure 8a, the number of L929 cells for all groups increased with the prolongation of culture time. In addition, the live/dead staining images of the cells are displayed in Figure 8b. Most cells were live (green dots), and almost no dead cells (red dots) were seen after culturing for 3 days.

The migration of L929 cells was also evaluated using a scratch-wound assay. First, thermal images of the wells in 6-well plates for different samples after irradiation with NIR light for 30 min were taken. As shown in Figure 8c, the temperature of the blank, PLLA, and CN/PLLA groups was <42°C. However, the temperature of the CN-MnO_2_/PLLA wound dressing reached 42.9°C, which is located in the range of mPTT (42–44°C). The wound healing images of L929 cells at different times are shown in Figure 8d and e. The wound distance in all groups decreased when the culture time ranged from 0 to 24 h. The wound distance in the CN-MnO_2_/PLLA group was smaller than that in the other groups regardless of irradiation with NIR light. There was no significant difference in the wound distance among the blank, PLLA, and CN/PLLA groups regardless of irradiation with NIR light. However, the wound distance in the CN-MnO_2_/PLLA group with NIR light was evidently smaller than that in the CN-MnO_2_/PLLA group without NIR light. The quantitative results of the wound area are shown in Figure 8f and g. The wound area in the blank, PLLA, and CN/PLLA groups with or without NIR light irradiation was 60–80% when further cultured for 24 h. For the CN-MnO_2_/PLLA group, the wound area after irradiation with NIR light was significantly reduced in contrast to that without NIR light. After coincubation for 24 h, the wound area in the CN-MnO_2_/PLLA group (26.7%) was much smaller than that in the blank (78.9%), PLLA (65.7%), and CN/PLLA (57.8%) groups after irradiation with NIR light. The scratch assay proved that the NIR light-treated CN-MnO_2_/PLLA group could better promote the migration of L929 cells, which was beneficial for wound healing.

### *In vivo* wound healing

**Figure 9:**
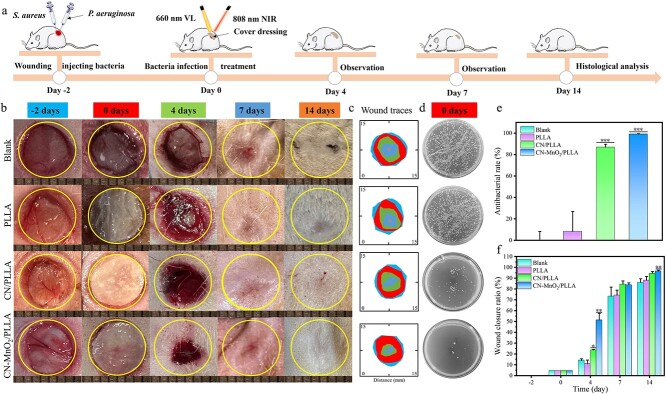
Detection of infectious wound healing rate *in vivo*. (**a**) Schematic diagram illustrating the timeline of the model fabrication of infectious skin wounds caused by *S. aureus* and *P. aeruginosa*, and experimental procedures. (**b**) Wound images (scale bar: 8 mm), (**c**) wound traces, and (**d**) bacterial colonies in the infectious skin wounds after implantation with different groups (blank, PLLA, CN/PLLA, and CN-MnO_2_/PLLA) for 4, 7, and 14 days under NIR light irradiation. (**e**) Antibacterial rate *in vivo* on Day 0 of different groups (blank, PLLA, CN/PLLA, and CN-MnO_2_/PLLA). (**f**) Wound closure rates of different groups (blank, PLLA, CN/PLLA, and CN-MnO_2_/PLLA) at Days −2, 0, 4, 7, and 14 were quantitatively analyzed*.* The significant difference represent ^*^p < 0.05, ^*^^*^p < 0.01, ^*^^*^^*^p < 0.001 compare with blank. *S. aureus staphylococcus aureus, P. aeruginosa pseudomonas aeruginosa, VL* visible light, *NIR* near-infrared, *PLLA* poly-L-lactic acid, *CN* graphitic phase carbon nitride, *MnO_2_* manganese dioxide

Inspired by the striking antibiofilm ability of CN-MnO_2_/PLLA, a skin defect model coinfected with *S. aureus* and *P. aeruginosa* was built on the backs of SD rats. On Day 0, the dressings were implanted in the infected skin wound areas. The timeline for model construction, treatment, and evaluation *in vivo* is shown in Figure 9a. Two days after surgery, the wound dressings were implanted in the skin defect sites. Moreover, the implantation sites were further irradiated with VL and NIR dual lights for 30 min. Figure 9b shows photographs of the infected wounds in the different groups at −2, 0, 4, 7, and 14 days. The wound area in all groups gradually decreased over time. After implantation for 4 days, the wound area in the CN-MnO_2_/PLLA group was significantly smaller than that in the other groups. On Day 14, the wounds in all the groups almost completely closed. Noticeably, no scar was found in the CN-MnO_2_/PLLA group on Day 14. The healing progress of the infected wounds was identified by measuring the area of the wounds within 14 days (Figure 9c). The healing rate of the CN-MnO_2_/PLLA group was faster than that of the other groups. This may be attributed to the elimination of persistent bacterial biofilm infection by the CN-MnO_2_/PLLA wound dressing, which promoted rapid healing of the infected wounds. After 14 days, the wound closure ratio in the CN-MnO_2_/PLLA group was almost 100%, while the wound closure ratios in the blank, PLLA, and CN/PLLA groups were ~86, 88 and 94.4%, respectively, and the results demonstrated that the CN-MnO_2_/PLLA wound dressing evidently accelerated infectious wound healing.

To evaluate the antibacterial effect of the wound dressings *in vivo*, tissue fluids from the wounds were collected after dual-light irradiation for 30 min. Then, the tissue fluids containing bacteria were cultured on agar plates (Figure 9d). Moreover, the antibacterial rate was calculated (Figure 9e). The results showed that few bacterial colonies were observed in the CN-MnO_2_/PLLA group, and the antibacterial rate was >90%. In contrast, a large number of bacterial colonies were present in the blank and PLLA groups (Figure 9d and e), indicating that the CN-MnO_2_/PLLA wound dressing had an efficient antibacterial effect *in vivo* under dual-light irradiation.

**Figure 10:**
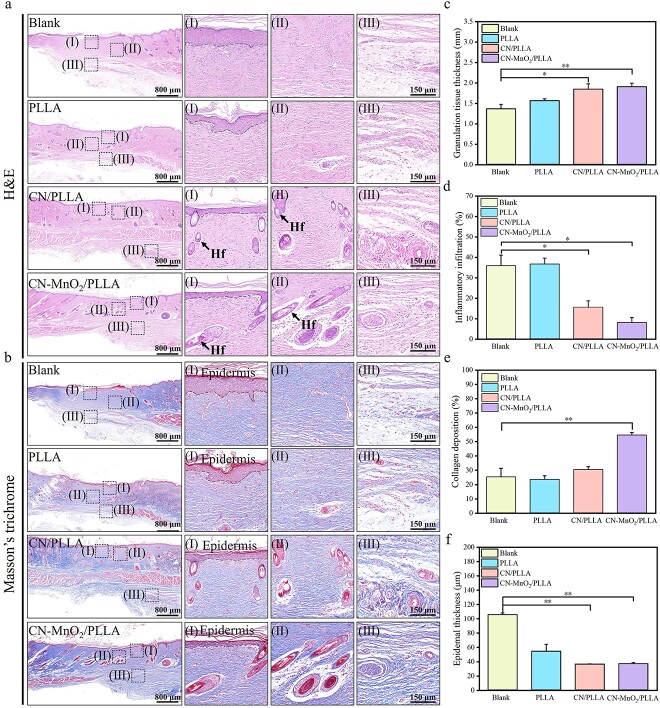
Representative H&E (**a**) and Masson’s trichrome (**b**) staining images of skin wound tissue in different groups (blank, PLLA, CN/PLLA, and CN-MnO_2_/PLLA) on Day 14 (scale bar: 800 μm) along with local magnification of the epidermis (I), dermis (II), and subcutaneous tissue (III) (Hf, scale bar: 150 μm). Semiquantitative analysis of (**c**) granulation tissue thickness, (**d**) inflammatory infiltration, (**e**) collagen deposition, and (**f**) epidermal thickness. Representative images were taken from five independent samples (*n* = 5). The significant difference represent ^*^p < 0.05, ^*^^*^p < 0.01 compare with blank. *Hf* hair follicle, *H&E* hematoxylin and eosin, *PLLA* poly-L-lactic acid, *CN* graphitic phase carbon nitride, *MnO_2_* manganese dioxide

After 14 days of implantation, the skin wound tissues were further evaluated via histological analysis and immunofluorescence staining. Representative H&E and Masson’s trichrome staining images of skin wound tissues are shown in Figure 10a and b, respectively. An obvious increase in the thickness of the granulation tissue at implantation sites covered by CN-MnO_2_/PLLA was observed (Figure 10c). There was substantial neutrophil infiltration in the blank and PLLA groups, which is a typical inflammatory response (Figure 10d). In particular, hair follicles (indicated by dark arrows) and few inflammatory cells were observed in the CN-MnO_2_/PLLA group (Figure 10a). Collagen deposition plays a crucial role in promoting wound healing. According to the Masson’s trichrome staining results shown in Figure 10b and e, the wound area in the CN-MnO_2_/PLLA group had more collagen deposition, an intact fiber structure, and a wavily arrayed structure compared to the other groups. Notably, the epidermal thickness in the CN-MnO_2_/PLLA group was the thinnest, indicating that the whole repair process from proliferation to remolding was being accelerated by CN-MnO_2_/PLLA and dual-light irradiation. Therefore, the above histological results indicate that the CN-MnO_2_/PLLA dressing significantly promotes rapid healing of infected skin wounds.

**Figure 11:**
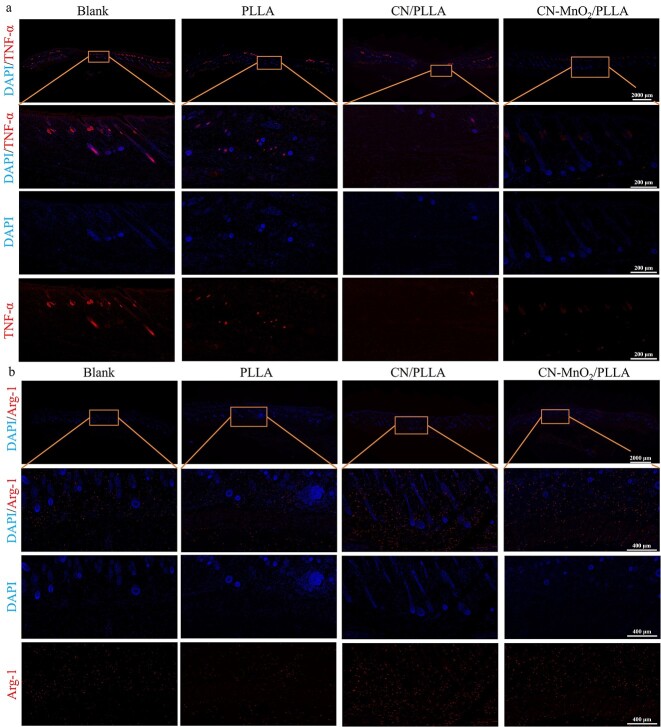
The expression of TNF-α and Arg-1 in *vivo*. (**a**) Immunofluorescence images of TNF-α in wound tissue of different groups (blank, PLLA, CN/PLLA, and CN-MnO_2_/PLLA). Scale bars: 2000 and 200 μm as indicated. (**b**) Immunofluorescence images of Arg-1 in wound tissue of different groups (blank, PLLA, CN/PLLA, and CN-MnO_2_/PLLA). Scale bars: 2000 and 400 μm. *DAPI* 4′,6-diamidino-2-phenylindole, *TNF-α* tumor necrosis factor-α, *Arg-1* Arginase 1, *PLLA* poly-L-lactic acid, *CN* graphitic phase carbon nitride, *MnO_2_* manganese dioxide

The anti-inflammatory effects on skin wound tissues were further evaluated by immunofluorescence staining. The signals of the pro-inflammatory marker (TNF-α) in the CN/PLLA and CN-MnO_2_/PLLA groups were significantly lower than those in the blank and PLLA groups (Figure 11a). However, the levels of anti-inflammatory marker (Arg-1) in the CN/PLLA and CN-MnO_2_/PLLA groups were evidently upregulated compared with those in the blank and PLLA groups (Figure 11b). In addition, the CN-MnO_2_/PLLA group displayed the lowest pro-inflammatory and the highest anti-inflammatory signals among these groups, suggesting that the inflammatory level was significantly ameliorated after CN-MnO_2_/PLLA and dual-light treatment (Figure 11a and b).

**Figure 12:**
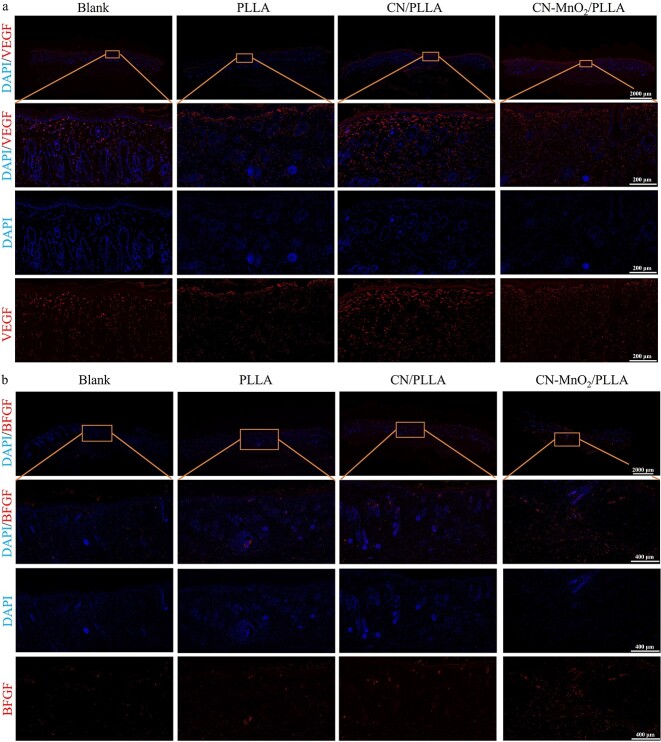
The expression VEGF and BFGF *in vivo*. (**a**) Immunofluorescence images of VEGF in wound tissue of different groups (blank, PLLA, CN/PLLA, and CN-MnO_2_/PLLA). Scale bars: 2000 and 200 μm as indicated. (**b**) Immunofluorescence images of BFGF in wound tissue of different groups (blank, PLLA, CN/PLLA, and CN-MnO_2_/PLLA). Scale bars: 2000 and 400 μm. *DAPI* 4′,6-diamidino-2-phenylindole, *VEGF* vascular endothelial growth factor, *BFGF* basic fibroblast growth factor, *PLLA* poly-L-lactic acid, *CN* graphitic phase carbon nitride, *MnO_2_* manganese dioxide

Immunofluorescence staining of VEGF in the wound areas was performed to assess neovascularization. On Day 14, the levels of VEGF in the wound areas in the CN/PLLA and CN-MnO_2_/PLLA groups were significantly greater than those in the other groups (Figure 12a). Moreover, the CN-MnO_2_/PLLA group exhibited the highest VEGF level. BFGF is an important indicator of wound healing that mediates macrophage and fibroblast chemotaxis toward the wound and promotes collagen synthesis [37]. As shown in Figure 12b, the expression of BFGF in the CN-MnO_2_/PLLA group was greater than that in the other groups.

## Discussion

Skin wound healing is susceptible to bacterial biofilm infections which inhibit wound healing. Novel antibacterial materials (such as antibacterial peptides, heavy-metal ions, etc.) have been applied in antibacterial research [5]. However, their expensive price and biological toxicity have constrained the wide application of antibacterial materials. PDT is a highly promising antibacterial therapy that utilizes photosensitizers to generate ROS such as ^1^O_2_, •OH, and ${\mathrm{O}}_2^{-}$ upon exposure to a specific wavelength of irradiation. PDT is widely noted for its less harmful effects on tissues during killing of bacteria. In many photodynamic agents, CN has good stability and biocompatibility. Therefore, it has great application prospects in PDT treatment of infected wounds. However, during the formation of bacterial biofilm, the generation of ROS within the bacterial biofilm is limited due to the low-O_2_ environment. Moreover, due to the formation of bacterial biofilm, ROS cannot enter the bacteria, thus affecting the bactericidal effect of ROS. The combination of photodynamic/photothermal therapy, photothermal/gas therapy, and so on, can offset the shortcomings of the single treatment modality to eliminate the biofilm efficiently. [38,39]. In this study, MnO_2_, which has enzyme-like activity, was grown on the CN surface to construct CN-MnO_2_ with enzyme-like activity. CN-MnO_2_ was utilized to convert H_2_O_2_ in the biofilm into O_2_, which provides the reaction raw material for photosensitizer CN. Meanwhile, the mPTT effect of MnO_2_ can improve the biofilm permeability and promote the entry of ROS into the bacteria.

As shown in the absorption spectra (Figure 1l), the absorption range of CN-MnO_2_ was significantly red-shifted by *in situ* growth of MnO_2_ on the CN surface, which effectively extended the optically absorbable region of CN from the visible region to the NIR region [40]. The 808 nm NIR light wavelengths can be absorbed by CN-MnO_2_.

As shown in Figure 2g, the porosity of the CN-MnO_2_/PLLA wound dressing was >90%. The high porosity of the CN-MnO_2_/PLLA not only enhances permeability and facilitates the exchange of O_2_, but also accelerates metabolism [41]. Moreover, high porosity also contributes to excellent moisture retention and effectively prevent wounds from becoming excessively dry, thereby enhancing the regenerative repair process of epithelial cells [42]. As shown in Figure 2h, the WVTR of CN-MnO_2_/PLLA wound dressing is within the normal range. Suitable permeability is beneficial to keep a moist environment on the wound surface. If the wound dressing could not offer adequate permeability, it might cause the skin wound to excessively dry out, thereby potentially impeding the healing process [43]. Therefore, the suitable WVTR of CN-MnO_2_/PLLA wound dressing is beneficial to wound healing. As shown in Figure 2i, the WCA of the CN-MnO_2_/PLLA wound dressing was significantly smaller than that of the PLLA wound dressing. WCA is an important indicator for evaluating the hydrophilicity or hydrophobicity of a solid surface. The small WCA indicates that the fluid is more easily spread over the CN-MnO_2_/PLLA dressing surface, thus benefiting cell growth and tissue repair [44,45].

Figure 3 tested the catalase activity and GPx activity of CN-MnO_2_/PLLA wound dressing. As shown in Figure 3b–f, the CN-MnO_2_/PLLA wound dressing was able to consume more H_2_O_2_, produce more O_2_, and exhibit more catalase activity at low pH. This experiment simulated the catalase activity of CN-MnO_2_/PLLA wound dressing under biofilm infection, and CN-MnO_2_/PLLA wound dressing exhibited higher catalase activity, which is favorable for generating O_2_ and solving the hypoxia problem. As shown in Figure 3g–m, the CN-MnO_2_/PLLA wound dressing exhibited high GPx activity and demonstrated a GSH consumption rate of almost 100% under dual-light irradiation. Encouragingly, the CN-MnO_2_/PLLA wound dressing showed 73 and 90% depletion of GSH within *S. aureus* and *P. aeruginosa*, respectively. Thus, the CN-MnO_2_/PLLA wound dressing could enhance ROS antibacterial activity by weakening the antioxidant system of bacteria. Therefore, testing the catalase activity and GPx activity of the CN-MnO_2_/PLLA wound dressing could be used to verify that the CN-MnO_2_/PLLA wound dressing could provide the reaction raw materials for the subsequent photodynamic experiments and weaken the antioxidant capacity within the bacteria to enhance photodynamic antibacterial therapy.

Ma et al prepared a kind of porous sheet-structured β-Bi_2_O_3_ with abundant oxygen vacancies, which achieved antibacterial properties via ROS [46]. Therefore, it is important to test the ROS production of CN-MnO_2_/PLLA wound dressings. Figure 4 tested the ^1^O_2_, •OH, and mPTT properties of the wound dressings under VL, NIR, and dual-light irradiation, respectively. As shown in Figure 4a–f, the CN-MnO_2_/PLLA wound dressing produced ROS under VL light irradiation, and more ROS were produced with the addition of H_2_O_2_. The results indicated that the generation of O_2_ through the consumption of H_2_O_2_ promotes the production of more ROS. As shown in Figure 4g–i, the CN-MnO_2_/PLLA wound dressing showed a mild temperature increase under NIR light irradiation. The experimental results indicated that extending the optically absorbable region of CN from the visible region to the NIR region by *in situ* growth of MnO_2_ on the CN surface provides the mPTT properties of the CN-MnO_2_/PLLA wound dressing.

As shown in Figure 5, the CN-MnO_2_/PLLA wound dressing can disrupt the complete bacterial biofilm under dual-light irradiation. Generally, biofilms are composed of various polysaccharides and proteins that protect bacteria from the negative effects of the surrounding environment [47,48]. In addition, the low O_2_ environment within the bacterial biofilms restricts the efficient conversion of O_2_ into ^1^O_2_ by photosensitizers [49–51]. The possible reasons that CN-MnO_2_/PLLA wound dressing could disrupt the integrity of bacterial biofilm are as follows. On one hand, CN-MnO_2_/PLLA wound dressing provided more O_2_ to alleviate the hypoxia within the biofilm and enhanced ROS production under the irradiation of VL light. On the other hand, the mild temperature generated by the CN-MnO_2_/PLLA wound dressing under the irradiation of NIR light was able to change the permeability of bacterial biofilm.

As shown in Figure 6, the CN-MnO_2_/PLLA wound dressing could achieve high antibacterial efficiency under dual-light irradiation, with antibacterial rates of 99 and 98.7% against *S. aureus* and *P. aeruginosa*, respectively. It can be observed that the CN-MnO_2_/PLLA wound dressing effectively eliminated a significant number of bacteria through synergistic PDT and mPTT effects. This validates the potential of elevated temperature to modulate the permeability of bacterial biofilms, catalase to mitigate hypoxia within them, and GPx to comsume the GSH within bacteria, thereby enhancing the antibacterial efficacy of PDT.

ONPG hydrolysis, lysozyme sensitivity, protein leakage, and ROS content inside the bacteria were further tested. As shown in Figure 7, the CN-MnO_2_/PLLA wound dressing increased ROS penetration into the bacterial interior by enhancing the permeability of the bacterial inner membrane, resulting in protein leakage and subsequent bacterial death. The antibacterial mechanism is similar to the heterojunction of zinc atom-doped g-C_3_N_4_ and Bi_2_S_3_ in a nanorod (CN–Zn/BiS), which demonstrated antibacterial properties by generating PDT and PTT to induce membrane polarization, ATP metabolic disorders, and protein or DNA/RNA release under NIR irradiation [39]. The results indicate that CN-MnO_2_/PLLA wound dressing synergistically alters the internal homeostasis of bacteria through PDT and mPTT, ultimately causing bacterial death.

Inspired by the outstanding *in vitro* results, an *in vivo* infected wound model was constructed. As shown in Figure 9, CN-MnO_2_/PLLA wound dressing could quickly promote wound healing and kill bacteria from the skin surface. This result showed that the elimination of bacterial infection is beneficial to wound healing.

In order to investigate the relationship between killing bacteria and promoting skin wound healing, H&E, Masson’ trichrome staining, the expression of pro-inflammatory cytokines, anti-inflammatory cytokines, VEGF, and BFGF in skin tissue slices were tested. As shown in Figs 10–12. It can be seen that there were no obvious inflammatory cells and more granulation tissue on the CN-MnO_2_/PLLA group (Figure 10a, c and d). As shown in Figure 10b, e and f, the CN-MnO_2_/PLLA group had obvious collagen deposition and thin epidermal thickness. Skin tissue repair generally experiences the following phases: coagulation, inflammation, proliferation and remodeling [52]. The results in Figure 10 illustrate that the CN-MnO_2_/PLLA group accelerated the whole repair process from proliferation to remodeling under dual-light irradiation, indicating that the CN-MnO_2_/PLLA group shortened the inflammatory phase by killing bacteria and thus promoting wound healing. As shown in Figs 11 and 12, the CN-MnO_2_/PLLA group achieved the promotion of wound healing by inhibiting excessive inflammation, and promoting vascularization and epithelization.

These findings indicate that the CN-MnO_2_/PLLA wound dressing effectively mitigates the hypoxic environment within the biofilm, depletes GSH within bacteria, and enhances ROS penetration into bacteria through elevated temperatures, thereby facilitating bacterial eradication on infected wounds. Meanwhile, CN-MnO_2_/PLLA also inhibits the expression of pro-inflammatory cytokines and promotes the expression of anti-inflammatory cytokines, vasculogenic cytokines and epidermal cytokines to promote rapid healing of infected wounds. Therefore, the CN-MnO_2_/PLLA wound dressing shows promise for application in the treatment of infected wounds.

## Conclusions

In this study, we synthesized CN-MnO_2_ nanozymes by growing MnO_2_ nanoparticles on the surface of CN. The nanozyme was then added to poly PLLA to fabricate a wound dressing using the electrospinning technique. Compared to pure CN, the CN-MnO_2_ nanoenzyme possesses both photodynamic and photothermal effects under VL and NIR light irradiation, respectively. Additionally, CN-MnO_2_/PLLA is able to generate O_2_ within bacterial biofilms, leading to increased production of ROS by consuming H_2_O_2_ through catalase and GPx. The CN-MnO_2_/PLLA dressing showed impressive antibacterial properties, achieving biofilm resistance rates of 83 and 62% against *S. aureus* and *P. aeruginosa*, respectively, with an overall antibacterial rate of 99%. The *in vivo* rat infection wound results showed that the CN-MnO_2_/PLLA dressing not only inhibited the growth of *S. aureus* and *P. aeruginosa*, but also significantly accelerated the wound healing rate by downregulating the expression of pro-inflammatory cytokines (TNF-α) and upregulating the expression of anti-inflammatory cytokines (Arg-1). Moreover, the CN-MnO_2_/PLLA dressing also promoted vascularization and epithelization. In conclusion, the prepared CN-MnO_2_/PLLA dressing shows promising potential for treating infected wounds.

## Abbreviations

Arg-1:Arginase 1; BFGF: Basic fibroblast growth factor; CCK-8: Cell counting kit-8; CN: Graphitic phase carbon nitride; CV: Crystal violet; DI: Deionized; DMEM: Dulbecco’s modified Eagle’s medium; DMSO: Dimethylsulfoxide; DPBF: 1,3-Diphenylisobenzofuran; DTNB: 5,5′ Dithiodithio 2-nitrobenzoic acid; EP tube: Eppendorf tube; FTIR: Fourier-transform infrared spectrometry; GPx: Glutathione peroxidase; GSSG: Oxidized glutathione; H&E: Hematoxylin and eosin; LB: Luria–Bertani; MB: Methylene blue; mPTT: Mild photothermal therapy; NIR: Near-infrared; OD: Optical density; ^1^O_2_: Singlet oxygen; •OH: Hydroxyl radical; ${\mathrm{O}}_2^{-}$: Superoxide anion; ATCC: American Type Culture Collection; CFU: colony forming units; ONPG: *o*-Nitrophenyl-β-D-galactopyranoside; DCFH-DA: 2′,7′-Dichlorodihydrofluorescein diacetate; PBS: Phosphate buffer solution; OD: optical density; std: standard deviation; DAPI: 4′,6-diamidino-2-phenylindole; EDTA: Ethylenediaminetetraacetic acid; ANOVA: Analysis of Variance; PDT: Photodynamic therapy; PLLA: poly-L-lactic acid; ROS: Reactive oxygen species; SD: Sprague–Dawley; SEM: Scanning electron microscopy; TEM: Transmission electron microscope; TMAOH: Tetramethylammonium hydroxide; TMB: 3,3′,5,5′-Tetramethylbenzidine; TNB: 2-Nitro-5-thiobenzoic acid; TNF-α: Tumor necrosis factor-α; UV: Ultraviolet; VEGF: Vascular endothelial growth factor; VL: Visible light; WVTR: Water vapor transmittance rate; WCA: Water contact angle; XRD: X-Ray diffraction; XPS: X-Ray photoelectron spectroscopy.

## Date availability

The data generated during the current study are available from the corresponding author on reasonable request.
